# Diversity of Bioinspired Hydrogels: From Structure to Applications

**DOI:** 10.3390/gels9050376

**Published:** 2023-05-02

**Authors:** Alexandra Lupu, Luiza Madalina Gradinaru, Vasile Robert Gradinaru, Maria Bercea

**Affiliations:** 1 “Petru Poni” Institute of Macromolecular Chemistry, 41-A Grigore Ghica Voda Alley, 700487 Iasi, Romania; 2 Faculty of Chemistry, “Alexandru Ioan Cuza” University, 11 Carol I Bd., 700506 Iasi, Romania

**Keywords:** hydrogel, biomolecule, polysaccharide, protein, peptide, biocompatibility

## Abstract

Hydrogels are three-dimensional networks with a variety of structures and functions that have a remarkable ability to absorb huge amounts of water or biological fluids. They can incorporate active compounds and release them in a controlled manner. Hydrogels can also be designed to be sensitive to external stimuli: temperature, pH, ionic strength, electrical or magnetic stimuli, specific molecules, etc. Alternative methods for the development of various hydrogels have been outlined in the literature over time. Some hydrogels are toxic and therefore are avoided when obtaining biomaterials, pharmaceuticals, or therapeutic products. Nature is a permanent source of inspiration for new structures and new functionalities of more and more competitive materials. Natural compounds present a series of physico-chemical and biological characteristics suitable for biomaterials, such as biocompatibility, antimicrobial properties, biodegradability, and nontoxicity. Thus, they can generate microenvironments comparable to the intracellular or extracellular matrices in the human body. This paper discusses the main advantages of the presence of biomolecules (polysaccharides, proteins, and polypeptides) in hydrogels. Structural aspects induced by natural compounds and their specific properties are emphasized. The most suitable applications will be highlighted, including drug delivery, self-healing materials for regenerative medicine, cell culture, wound dressings, 3D bioprinting, foods, etc.

## 1. Introduction

Hydrogels are three-dimensional (3D) crosslinked networks with solid-like properties, able to retain a high amount of water or biological fluids and to maintain their structural and functional integrity in various environmental conditions or under the action of external stresses (temperature, mechanical forces, light, electric or magnetic field, etc.). The networks are generated by physical, chemical, dynamic chemical, or combined crosslinking methods [1,2,3]. The physical crosslinking can be provided by different intra- and intermolecular interactions, such as hydrogen bonding, van der Waals interactions, hydrophobic interactions, or physical entanglements, without covalent crosslinking. On the other hand, chemically crosslinked hydrogels are formed due to the covalent or ionic bonds between the polymeric chains, and this involves the use of some crosslinking agents (molecules that contain two or more reactive sites able to be attached to specific functional groups) [4,5,6,7].

Natural or synthetic macromolecules, as well as various combinations of them, can be used to create hydrogels [6,7,8,9,10,11,12,13]. Hydrogels prepared from natural macromolecules, including polysaccharides and proteins, are suitable for a variety of applications due to their biochemical similarity with the human extracellular matrix (ECM). An additional benefit is that degradation products are recognized and further metabolized by the body. They present desirable characteristics for biomaterial design, such as low toxicity, biocompatibility, biodegradability, and availability, which are essential for biomedical- and therapeutic-related applications [11,12,13,14,15]. Hydrogels that include synthetic compounds present the advantages of control over the synthesis processes, definite molecular weights and chemical structures, and good mechanical properties. However, they have limited biocompatibility and biodegradability compared to natural polymers [2].

Different sources for extraction of natural polymers have been reported in the literature [16,17]. Most of these polymers come from sources such as animals, plants, and algae, but alternative biopolymers can also be obtained through microbial or enzymatic processes [18]. Polysaccharides are widely used for biomedical applications because of their biocompatible nature, diverse architectures, and ability to form gels in well-defined conditions [2,15]. Of particular interest are amphiphilic polysaccharides able to develop hydrophobic associations in self-healing hydrogels that recover their original structure and properties after the action of an external stress [19].

On the other hand, peptides are relatively easily synthesized and, in some cases, manufactured. As building blocks for various biomaterials, peptides have several advantages: they are composed of nonpolar, polar, or charged amino acids, allowing a certain level of prediction of self-assembly properties through the meticulous selection of the peptide sequence [20]. As endogenous molecules, they reduce the risk of adverse effects. They can range from short to long and more flexible chains, ensuring the construction of diverse structural arrangements, from solid crystals to soft, disordered materials. Furthermore, peptides can generate smart materials responsive to external stimuli such as temperature, pH, or the presence of specific molecules.

Different biomimetic materials are continuously designed using native, modified, and synthetic (macro)molecules as building blocks. These materials are inspired by the biological role of polysaccharides, proteins, and peptides in nature. A challenge assumed by researchers is the design of novel peptide/polymer materials with targeted properties [21]. The novel strategies involve either the design of peptide amphiphiles or the replication of protein secondary structural motifs, such as α-helix and β-sheet. Many efforts have been focused on peptides folding into β-sheets (amyloid-like structures [22]) and also on the rational design of α-helical folding (proteins/peptide stabilization) [23].

Over the years, scientists have improved some chemical and physical features of hydrogels, such as self-healing ability, viscosity, and physical strength in order to enhance their biofunctionality and to extend their applicability [24,25]. The conjugation of bio-macromolecules with synthetic or natural polymers gives a diverse and broad class of materials [26].

**Scheme 1:**
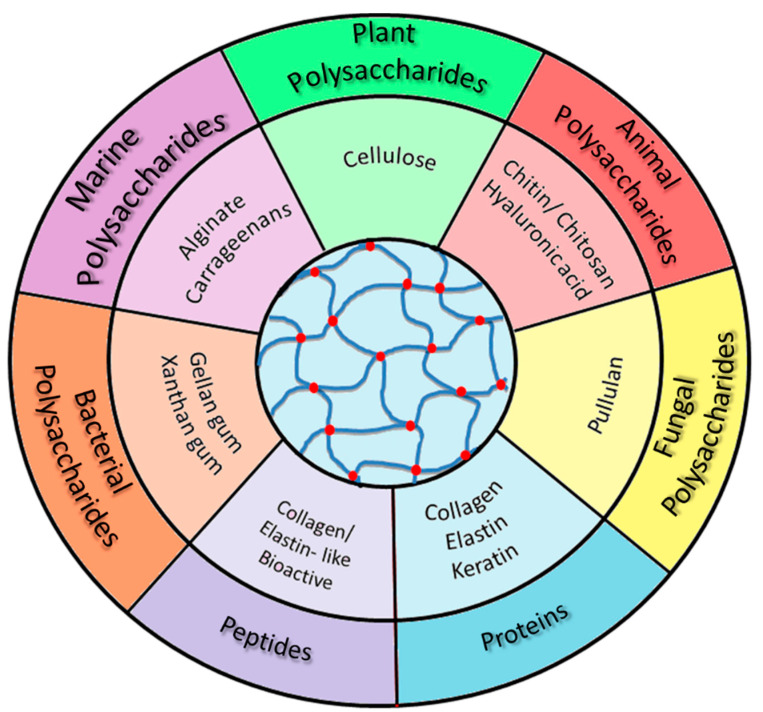
The main types of biomolecules used in the development of hydrogels (discussed in the present paper).

Polysaccharides and proteins/peptides present valuable properties that make them suitable for various challenging applications. The paper aims to briefly present a selection of the most significant natural compounds used in the design of numerous cutting-edge materials with applications in various fields. The discussion focuses on hydrogels based on natural polymers associated with active compounds with physiological implications and applicability. Thus, chitosan and its derivatives, hyaluronic acid, cellulose, gellan gum, xanthan gum, alginate, carrageenans, and pullulan are among the main polysaccharides that will be discussed in this paper (Scheme 1). At the same time, other biocompounds, including proteins and peptides, will be addressed.

## 2. Polysaccharides—From Structural Aspects to Multifunctional Hydrogels

Carbohydrates, also denoted as sugars, are biomolecules that exist in abundance in nature, with monosaccharides as the basic structural units, including from three to nine carbons and a carbonyl group (aldehyde in aldoses or ketone in ketoses). Cyclic monosaccharides are linked via α or β glycosidic bonds resulting in linear or branched chains with carbon, hydrogen, and oxygen, usually in the ratio of 1:2:1, (CH_2_O)*n*: for *n* < 20 the structures are denoted as oligosaccharides; when *n* > 20 the long chains are considered polysaccharides. Among oligosaccharides, some trisaccharides are well known, such as maltotriose and nigerotriose—3 glucose units joined by α(1→4) or α(1→3) glycosidic links, respectively, maltotriulose (glucose-α(1→4)–glucose-α(1→4)-fructose) or raffinose (galactose-α(1→6)-glucose-α(1→2)fructose). Additionally, oligo- and polysaccharide chains, composed of monosaccharides linked by chemical bonds, are referred to as glycans. These glycans are present in cells, mediating various interactions (cell–cell, cell–matrix, and cell–molecules). Deep theoretical and experimental investigations allow a better understanding of carbohydrate structures and their specific interactions in various environments and self-assembling phenomena [16,27]. Modern techniques and new approaches are available for these studies, such as nuclear magnetic resonance (NMR) spectroscopy [27,28], atomic force microscopy (AFM) [29,30,31,32], infrared (IR) spectroscopy [33,34], liquid or gas chromatography [35,36,37,38], capillary electrophoresis [39,40,41], mass spectrometry [42,43,44], molecular modeling [31,45,46], or analytical techniques [47].

A high diversity of polysaccharides is found in nature with different types of glycosidic linkages, which induce unique physico-chemical characteristics and functional versatility [16,17,48]. From a structural point of view, a common building block is represented either by a pyranose or a furanose carbohydrate ring. According to their origin, polysaccharides can be grouped as follows [17,48,49] (Scheme 1):

− *Animal polysaccharides*: chitin (CT)/chitosan (CS), hyaluronic acid (HA), etc.;
− *Plant polysaccharides*: cellulose (CELL), starch, pectin, gum arabic, brea gum, etc.;
− *Bacterial polysaccharides*: gellan (GG), xanthan gum (XG), dextran (Dex), etc.;
− *Marine (algal) polysaccharides*: alginate (Alg), carrageenan (Carr), agar, agarose, etc.;
− *Fungal polysaccharides*: pullulan (PULL), scleroglucan, schizophyllan.

Some of these polysaccharides are discussed further. The structural aspects are briefly presented at the beginning of each section, and some recent research on polysaccharide-based hydrogels is then highlighted.

### 2.1. About Polysaccharide Chain Stiffness

The properties of polysaccharides, their gelation behavior, and specific hydrogel properties are strongly correlated with characteristic conformational aspects. Each polysaccharide has a peculiar structure that influences chain flexibility, usually determined in the unperturbed state [50,51,52]. For charged polysaccharides, it was found that a decrease of intrinsic viscosity ([*η*]) in aqueous solutions, caused by salt addition, depends considerably on the chain structure. From the dependence of [*η*] on the ionic strength, *I*, a dimensionless parameter *B* was simply defined, and it can be considered as a criterion for discussing a polysaccharide’s chain stiffness [53]. For water soluble polyelectrolytes, a correlation between *B* and Kuhn length was found [52]. Now, it is generally accepted that the Smidsrød–Haug parameter, *B*, quantifies the relative stiffness of biopolymers at fixed ionic strength, *I* (typically 0.1 M NaCl) [52,54,55,56].

**Figure 1:**
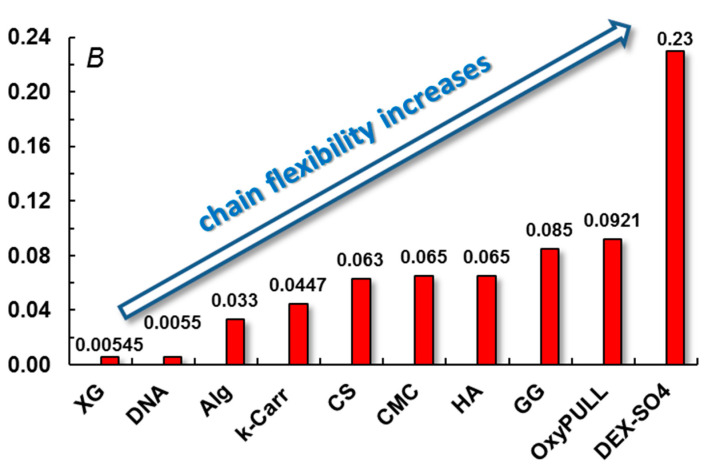
A comparative image of chain flexibility for different polysaccharides. *B* represents the stiffness parameter reported for polysaccharides at fixed ionic strength (0.1 M NaCl): xanthan gum (XG) [57], deoxyribonucleic acid (DNA) [53], alginate (Alg), M-block [52], κ-carrageenan (κ-Carr) [54], chitosan (CS) [58], carboxymethyl cellulose (CMC) [52], hyaluronic acid (HA) [52], gellan gum (GG) [52], oxidized pullulan (OxyPULL) [59], and dextran sulfate (DEX-SO4) [53]. Adapted with permission from [54], copyright 2019, Elsevier.

Figure 1 illustrates the chain flexibility (based on the stiffness parameter) for different polysaccharides discussed in this review.

According to values of the *B* parameter, XG helices [57,59] and mechanical inflexible DNA [53] present high stiffness. Chain stiffness decreases progressively for semiflexible Alg [53], k-Carr [54], CMC [52], or CS [58]. Flexibility increases for PULL, dextran, and their derivatives [53,60,61,62].

Pullulan and dextran chains in solution adopt various random coil conformations due to independent rotations around the bridge oxygens, and their backbone is very flexible (entropic driven) [40,60,61,62]. It was found that Alg flexibility is influenced by its structure, consisting of β-D-mannuronate (M-block) and α-L-guluronate (G-block) in different ratios. The unperturbed dimensions (influenced by the freedom of rotation around single bonds along the polymer chain and the geometry of the structural units) increase from MG-blocks to MM-blocks, and the highest values were obtained for GG-blocks [63]. Thus, the following *B* values were reported for different Alg structures: 0.033 for M-block and 0.038 for G-content of 0.36 and 0.065 for polyalternating structure [52,53,55,63]. XG [57,64,65], κ-carr [54,66,67], or GG [52,68] in solution present either ordered conformations, as single or double helices, or flexible coil conformation in disordered state. The adopted conformation is mainly influenced by temperature and ionic strength. These aspects will be discussed in the next sections.

### 2.2. Animal Polysaccharides

#### 2.2.1. Chitosan and Its Derivatives

Chitosan (CS) is a polysaccharide obtained by deacetylation of chitin, composed of glucosamine and N-acetyl-glucosamine units [69]. CS has been reported to be the second most abundant biopolymer in the world [70] and the most extensively used natural cationic biomaterial [71]. Due to the protonated primary amine groups, chitosan is soluble in low pH (acid) medium but insoluble in neutral or high pH (alkaline) solutions. Acetic, formic, hydrochloric, and lactic acids are among the most frequently used to improve the solubility of chitosan. Strong acids, such as sulfuric and phosphoric acid, are not recommended because of their degradative effect on chitosan chains [72]. In terms of properties, CS is a nontoxic, nonimmunogenic, biocompatible, and biodegradable biopolymer, widely recognized for its bacteriostatic, fungistatic, hemostatic, and antiulcerous activity [73,74,75,76].

Due to the low water solubility of chitosan, its methylated derivatives have been preferred when developing materials for healthcare industries [77,78]. Biological properties of carboxymethyl chitosan (CM) include antioxidant, antimicrobial, and apoptosis inhibitory activity. Therefore, they have been used in wound healing applications, cancer therapy, gene therapy, biosensors, and drug or bioactive component delivery [79,80,81].

**Figure 2:**
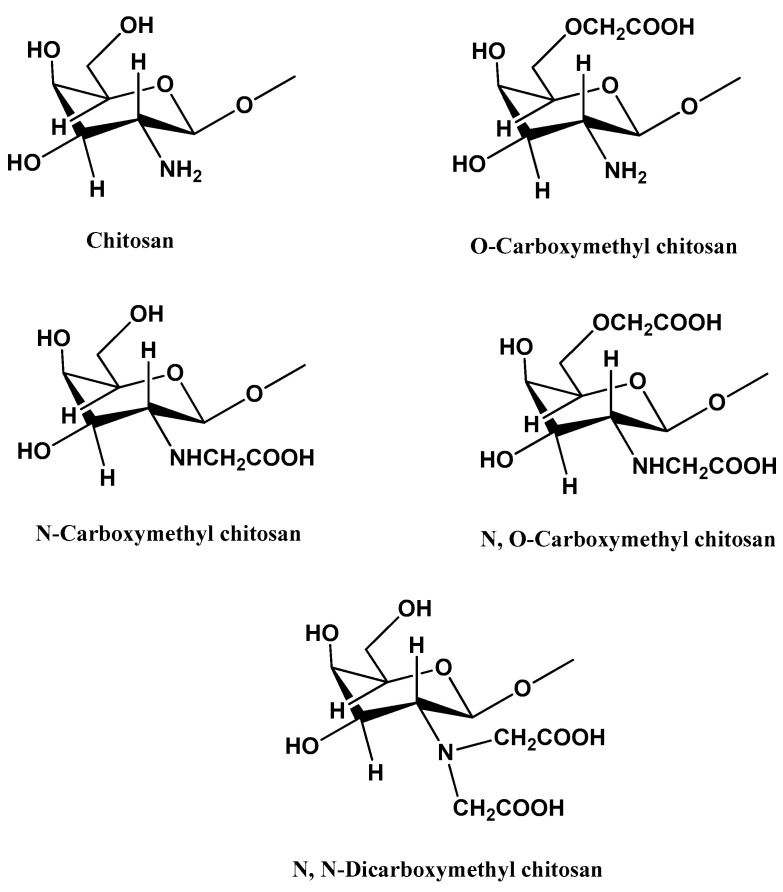
Structural representation of chitosan and carboxymethyl chitosan derivatives.

Figure 2 shows the structures of CS and some CM derivatives.

Being a cationic polysaccharide, the antimicrobial properties of chitosan are related to the electrostatic interactions between multiple negatively charged cell walls of some bacteria and its multiple positively charged chains [82]. The antimicrobial effect of chitosan and its derivatives against bacteria, yeasts, or filamentous fungi has been demonstrated in many studies [83,84]. For example, a recent study describes the synthesis of a chitosan-monoaldehyde-based hydrogel with antifungal properties for wound healing applications [85]. The hydrogels were highly porous with an average pore diameter of approx. 80 μm and a swelling rate controlled by the crosslinking density and medium pH. During 21 days of testing, the hydrogels showed a progressive weight loss in the presence of lysozyme up to 35%. They proved a noncytotoxic effect on normal human dermal fibroblasts using MTS test.

#### 2.2.2. Hyaluronic Acid and Derivatives

**Figure 3:**
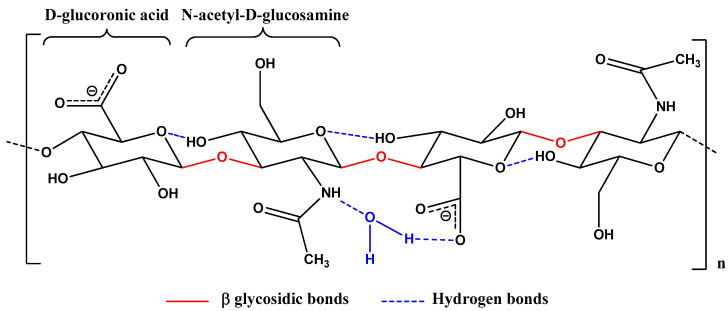
Chemical structure of hyaluronic acid. Hydrogen bonds are illustrated with blue; glycosidic bonds are represented with red.

Hyaluronic acid (HA) is a biodegradable and nontoxic heteropolysaccharide that is part of the glycosaminoglycan family and consists of repeated disaccharide units, β-1,4-D-glucuronic acid and β-1,3-N-acetyl-D-glucosamine. The molecule has a hydrophilic nature, with a simple, linear chemical structure maintained by glycosidic bonds and hydrogen bonds (Figure 3) [86]. HA presents suitable biophysical properties that naturally exist in the ECM, synovial fluid that surrounds cartilage, joints, and skin tissues [12,87], being able to mediate the biological and structural properties, such as matrix organization, wound repair, cellular signaling, or morphogenesis [88]. HA is synthesized by most cells in the human body. However, mesenchymal cells are believed to be the predominant HA source [86,89].

The molecular weight of HA varies within a large range, from oligosaccharides (<10^3^ g/mol) with low molecular weight (10^3^ g/mol–2.5 × 10^4^ g/mol) and medium length chains (2.5 × 10^4^ g/mol–10^6^ g/mol) to high molecular weight (>10^6^ g/mol) and very high molecular weight (>6 × 10^6^ g/mol) [90]. Depending on its molecular weight, HA displays different performances suitable for different applications. For example, high-molecular-weight HA presents an anti-inflammatory effect by stimulating the production and migration of cytokines, while very high-molecular-weight HA can affect the skin regeneration process by limiting the nutrient supply and inhibiting endothelial cell proliferation [91]. Molecular weight influences the viscosity, gelation time, elastic modulus, and molecular diffusion, as well as the degradation process. The optimal molecular weight of HA for in-situ-forming hydrogels was found around 5 × 10^5^ g/mol. This hydrogel promoted cell proliferation and in vitro vascular network formation [92].

Due to its anti-inflammatory, antiaging, and biocompatible properties, HA is used in a wide range of biomedical and cosmetic applications, such as skin and cartilage repair, tissue regeneration, wound healing, cell culture, 3D bioprinting, drug delivery, and various cosmetics [93,94,95,96,97,98]. Additionally, thanks to the functional groups in its structure, HA supports chemical changes to tailor physico-chemical properties for drug-carrier functions [86,99].

Viscosupplementation, also known as HA injection, represents a treatment for symptomatic osteoarthritis by improving the biomechanical functions of the tendons or/and joints. Intra-articular application can be used either as augmentation after surgical procedure, in order to treat osteochondral lesions, or as alternative to surgical interventions for patients with early phase osteoarthritis [100]. Besides HA injections, oral preparations of HA have been reported to lower pain and improve life quality of patients with osteoarthritis [101].

Unfortunately, they cause different side effects, including gastrointestinal reactions or renal dysfunction. Over the years, different materials have been developed for osteoarthritic therapy based on HA in combination with collagen, another natural component of ECM [102,103]. Another deficiency of HA hydrogels is low mechanical strength and susceptibility to degradation by hyaluronidase. To overcome such problems, various strategies have been used for structural modification, such as physical, chemical, or multiple crosslinking, as well as the introduction of reactive and functional molecules or moieties [93,104].

**Figure 4:**
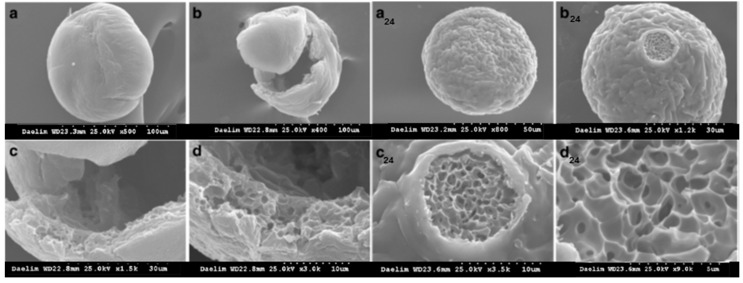
SEM images of crosslinked HA microspheres (**a**–**d**) for HA of 6.97 × 10^5^ g/mol. Images (**b**–**d**) show the hollow structure inside the microsphere. The index 24 refers to sample’s morphology after 24 h. Adapted from [105], https://www.ncbi.nlm.nih.gov/pmc/articles/PMC5013587/ (accessed on 26 April 2023).

Hyaluronic acid biphasic fillers containing crosslinked microspheres that were included in a solution of free HA chains were designed to correct facial defects [105]. The nanoparticle size decreases with increasing molecular weight of HA due to more preponderant intramolecular crosslinking as compared with an intermolecular one, determining a change of rheological behavior, i.e., a more pronounced fluid-like behavior. The viscoelastic moduli were tailored using an optimum chain length; for example, for a sample of 6.97 × 10^5^ g/mol, porous microspheres were obtained, and G’ ranging from 211 Pa to 420 Pa and G” was found between 129 Pa and 214 Pa, as a function of volume fraction of crosslinked HA, whereas particle texturing feel scaled between 7 and 9. In contrast, no pores on the surface of microspheres were observed from high molecular weight HA (>10^6^ g/mol) (Figure 4) [105].

Wound healing is also a common application for HA-based materials [106,107]. Recently, new hydrogels composed of chitosan derivatives (N-succinyl chitosan [45] or gallic-acid-grafted quaternized chitosan [107]) and oxidized hyaluronic acid were reported. Self-healing ability and flow behavior, as well as wound healing efficiency and in vivo hemostasis performances of these materials demonstrated great potential for wound healing applicability [106,107].

### 2.3. Plant Polysaccharides

#### Cellulose and Derivatives

**Figure 5:**
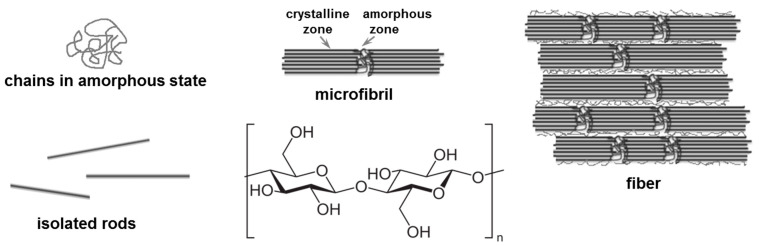
Structure of cellulose at different structural levels.

Cellulose (CELL) is the most abundant and relatively cheap renewable resource produced by plants, agro-residues, algae, tunicates, or bacteria [108]. CELL is a linear, semiflexible polymer, constituted from several hundred to over tens of thousands of glucose units, organized in crystalline and amorphous phases as microfibrils or fibers (Figure 5). The strong intra- and intermolecular hydrogen bonds at different length scales, Van der Waals and hydrophobic interactions [109] between cellulose chains, as well as the crystalline structure, give high stiffness and strength to native CELL and make this biopolymer insoluble in water or classical organic solvents. CELL is soluble in NaOH solutions [110], NaOH/urea, NaOH/thiourea solutions [111,112], and some green solvents, such as ionic liquids or deep eutectic solvents [111,113].

Intermolecular interactions at different structural levels are very complex, generating various packing arrangements in four crystalline allomorphs: cellulose I, II, III, and IV. The native form is cellulose I found in all plant cell walls, as a combination of two allomorphs: Ia and Ib. Cellulose II is obtained from cellulose I during an irreversible process of regeneration or alkali treatment. Cellulose III results at low temperatures from cellulose I or cellulose II in an ammoniacal aqueous solution or using an organic amine. Cellulose IV is obtained from thermal treatment of cellulose III in glycerol [109].

Cellulosic samples present both crystalline and amorphous domains, and their ratio depends on CELL provenience and sample history. The functionalization of CELL is strongly influenced by its structure, and, for chemical reactions, the available sites are either the amorphous regions or the surface of crystallites.

Very often, the term nanocellulose is used, and it refers to isolated anisotropic (crystalline) nanostructures obtained after alkaline treatment, bleaching, and hydrolysis (acid, enzymatic, or subcritical water hydrolysis) [114] applied to the raw biomass. The maximum yield of recovered nanocellulose from cellulose material is about 70–80%, corresponding to a total yield of 20–30% from the original bioresource [114]. Nanocellulose denotes either cellulose nanocrystal (CNC), cellulose nanofibers (CNF), or bacterial nanocellulose (cellulose produced by bacteria) with a high aspect ratio (length to width ratio from tens to hundreds).

CELL is an ideal source for preparing green hydrogels with excellent water absorption capacity, at relatively low cost. Due to difficulties related to its dissolution, the use of CELL is still limited. The most appropriate approach is its functionalization by modifying the -OH groups of the parent structure into ether (metyl-, ethyl-, hydroxyethyl-, hydroxypropyl methyl-, carboxymethyl-, or hydroxypropyl cellulose) or ester (cellulose acetate, cellulose acetate phthalate, or cellulose acetate butyrate) derivatives. Some of the most important ones are briefly presented below.

Hydroxypropyl methylcellulose (HPMC) is a thermosensitive, biocompatible derivative, able to form transparent hydrogels with good stability and viscoelasticity, suitable for the development of various biomaterials, such as scaffolds, films, or membranes [111]. It was recently used as a matrix component for improving the delivery of hydrophobic bioactive compounds, such as quercetin [115].

Hydroxyethyl cellulose (HEC) is a cellulose ether soluble in water mainly used for the fabrication of biocompatible and biodegradable hydrogels for wound dressing [116,117], 3D printing and wearable electronic devices [118], and in pharmaceuticals and cosmetics, as a stabilizer, thickener, or coating agent [111].

Carboxymethyl cellulose (CMC) presents carboxylic groups along the cellulosic backbone, which confers high water solubility and chemical reactivity. This derivative is an ideal candidate for obtaining hydrogels with high absorption in physiologically relevant solvents [119], as well as mechanical strength and biodegradability [120]. The high potential of CMC hydrogels in various applications was presented in recent papers [120,121,122,123,124]: wound dressings, foods, water decontamination by removal of toxic dyes, and agricultural applications (holding water in soil or targeted delivery of agrochemicals).

Hydroxypropyl cellulose (HPC) is a biocompatible cellulose derivative used in many applications, such as thickening or binding agents in pharmaceutics, foods, and cosmetics. HPC is soluble at low temperatures and develops hydrophobic interactions at increasing temperatures, a phase separation occurring above 40 °C. At room temperature, semidiluted and concentrated HPC aqueous solutions form an isotropic phase. Above a certain concentration, HPC chains are organized in an ordered liquid crystalline phase with a cholesteric structure. The elasticity of viscoelastic HPC fluids is attributed to a high extent to the orientational distortion of the director in the nematic phase, whereas the contributions of chain entropy and interfacial tension are considerably smaller [125]. HPC was used to prepare hydrogels that maintain adequate moisture for wound healing [126].

Due to the hierarchical structure of CELL, its materials present unique characteristics, such as high strength, low density, biocompatibility, and excellent mechanical and barrier properties. CELL-based systems are pseudoplastic and exhibit thixotropy, self-healing ability, and liquid crystalline properties [127,128,129,130,131,132,133,134,135]. These exceptional properties of CELL have attracted considerable interest not only for paper and boards, but also for design of new functional materials for a wide range of applications, including hydrogels; aerogels and coating films; 3D printing materials; drug-delivery vehicles; rheological modifiers; biomedical, healthcare, and food applications; oil and gas industry applicaitons; packaging; various environmentally friendly nanocomposites; hygiene and absorbent products; electronics; textiles; filtration; etc. High-performance materials based on nanocellulose or cellulose derivatives are a hot subject debated in a huge number of research articles and reviews (see for example [108,110,114,127,136,137,138,139,140,141]).

### 2.4. Bacterial Polysaccharides

#### 2.4.1. Gellan Gum

**Figure 6:**
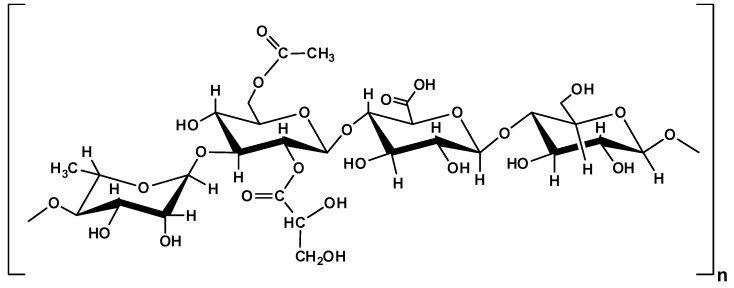
Chemical structure of gellan gum.

Gellan gum (GG) is produced through the microbial fermentation of Gram-negative *Sphingomonas elodea* bacteria in sugar-rich media. It is a linear, anionic polysaccharide based on a tetrasaccharide repeating unit (Figure 6), with an average molecular weight of about 5 × 10^5^ g/mol. In solution, GG chains form a three-fold double helix from two left-handed chains; the glyceryl groups stabilize the interchain associations through hydrogen bonds. In their native state, some acetate residues can be located on the periphery of the double helix [68,142,143,144].

**Figure 7:**
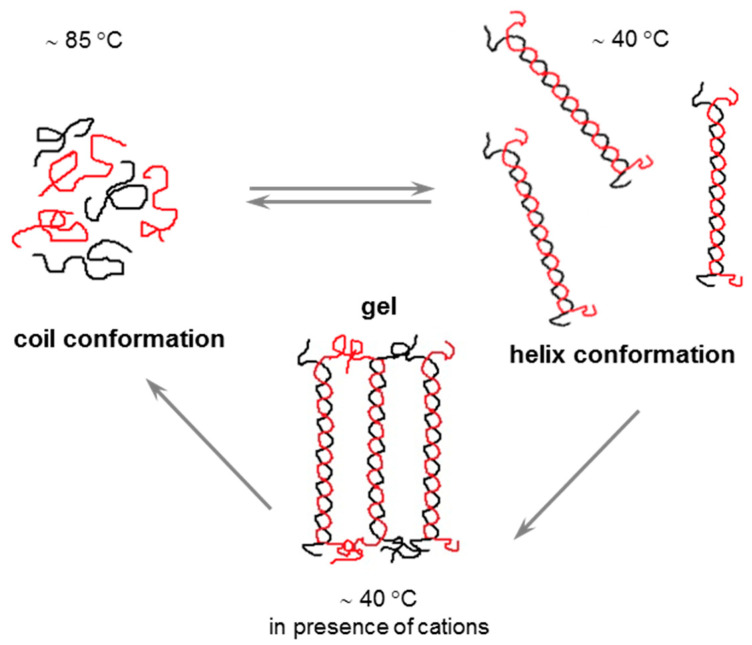
Schematic presentation of conformational changes and gelation of GG.

Gellan is one of the most studied biomolecules due to its ability to form stable networks by aggregation of double helices at temperatures below 40 °C, when divalent ions are added to its solution (Figure 7). Low pH and monovalent cations promote aggregation and gelation by decreasing the negative charges or by binding the double helices in coordination sites around carboxylate groups [68,143,144]. Furthermore, some anions in aqueous solutions are able to induce changes in the conformation of GG. A preferential affinity of I^−^ for GG ordered conformation was recently discussed [145].

The gelation of GG is thermoreversible and occurs around 40 °C through aggregation of double helices, being influenced by polysaccharide concentration [146] or nature and concentration of cations [147,148]. The mechanical and rheological properties of the GG gel are influenced by the degree of acylation: acetylated GG leads to elastic, flexible, and transparent soft gels [146,149], while low-acetyl or deacylated GG gives brittle gels. Their strength/hardness can be enhanced by microwave heating [150].

GG also forms hydrogels with multivalent cations, able to absorb a high amount of water or body fluids or to encapsulate drugs or cells in interstitial spaces among polymer chains involved in ordered structure [151,152].

Due to its peculiar properties, GG is mostly used as a suspending, stabilizing, thickening, and gelling agent in various applications: foods, pharmaceuticals, cosmetics, toiletries, biomedicine, tissue engineering, or microbiology [153,154].

The high number of hydroxyl and free carboxyl groups in each repeating unit makes GG a versatile component for improving the rheological and biological properties of materials [142,153,154]. GG-based gels or interpenetrated networks, obtained by using GG in the presence of other biomolecules, such as chitosan, sodium Alg, CELL, PULL, XG, agar, starch, gelatin, silk fibroin, etc. [143,153,155] or by functionalization [156,157] presented enhanced properties.

Various 3D printing strategies use GG for biofabrication of materials with complex shapes for tissue engineering applications [154]. It was shown that 10% gelatin-methacryloyl in the presence of 0.5% GG is a suitable system for cartilage bioprinting [158,159]. The addition of a small amount of GG (below 0.5% GG) enhanced the printability by inducing yield stress behavior, thus increasing the shape fidelity. High GG content (>0.5% GG) determines an increase of yield stress value and makes difficult the cell encapsulation [158]. The yield stress values, shear thinning, thixotropy, and viscoelastic behaviors of inks correlated with cell viability represent key characteristics for the bioprinting process [140]. Cell viability is negatively influenced by increased shear forces, and a preliminary rheological characterization is required for successful bioprinting [140,160].

Synergistic multicomponent polysaccharide hydrogels, such as Alg/GG/XG networks, present high swelling ability, consistency, and thermal stability, being of interest for the restructuring of foods or in nutraceutical delivery [161]. GG and locust bean gum (LBG) double networks with pH-sensitive LBG borate-ester bonds and hydrogen bonds between GG double helices showed shape memory, self-healing properties, and improved mechanical behavior as compared with single polysaccharide gels [162]. These materials are appropriate for soft robotics and biomedicine.

Gelation of low acyl GG in the presence of low methoxyl citrus pectin (30.4% methoxyl) provided more rigid hydrogels with narrow meshes (6.1–11.7 nm) and slower chain dynamics (relaxation time, λ, in the range 16.7–55.2 s) as compared with GG (λ from 18.4 s to 29.3 s). GG predominates in the junction zones and in the presence of Ca^2+^ pectin contributes to network enhancement through additional interactions. This system formed by biomolecules is suitable for biomedical applications, such as vehicles for drug delivery [163]. In the presence of calcium cations, a pH-sensitive gelling effect is observed for pectin solutions [164].

**Figure 8:**
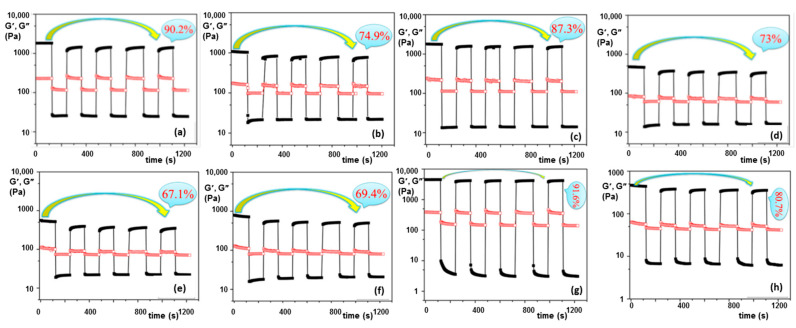
Illustration of self-healing ability of salecan/gellan gum hydrogels tested during cyclic deformation tests at low (0.1%) and high (100%) successive strain values (ω = 10 rad/s, 25 °C). The black symbols denote the elastic modulus, G’, and the red symbols represent the viscous modulus, G”. (**a**) S/GG = 1:1; (**b**) S/GG = 2:1; (**c**) S/GG = 1:2; (**d**) S/GG = 1:1; (**e**) S/GG = 2:1; (**f**) S/GG = 1:2; (**g**) 2% GG; (**h**) 1% GG; concentration: S—2% for all samples; (**a**–**c**,**g**) 2% GG; and (**d**–**f**,**h**) 1% GG. Adapted with permission from [165], copyright 2020, Elsevier.

The application of GG-based hydrogels has been extended in many fields, from vehicles for drug delivery to cell delivery for rejuvenating tissue [151]. Composite hydrogels with other biopolymers (such as salecan, S) were obtained from aqueous solutions using physical methods (in the presence of 10 mM CaCl_2_ and 0.25 M NaOH) [103]. Rheological investigations revealed the formation of elastic hydrogels, with yield stress, shear thinning behavior, and self-healing ability (Figure 8).

Short aromatic peptide derivatives, such as 9-fluorenylmethoxycarbonyl (Fmoc), and short peptides, such as Fmoc-Gly-Gly, self-assembled through hydrogen bonds with GG or agarose with the formation of viscoelastic gels, are suitable as substrates for cell cultures. These hybrid networks presented good cell viability on fibroblasts and in vivo biocompatibility [166]. It was recently shown that the encapsulation of anthocyanins with gelatin/GG gum avoids degradation and maximizes their release in the small intestine [167].

#### 2.4.2. Xanthan Gum

**Figure 9:**
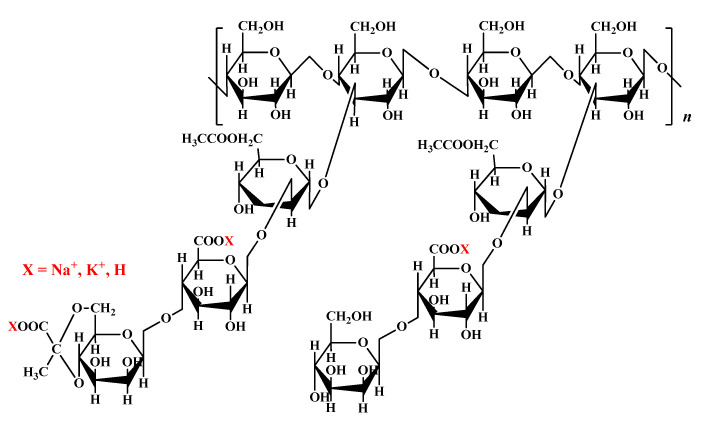
Chemical structure of xanthan gum.

Xanthan gum (XG) is a microbial exopolysaccharide produced by Gram-negative bacteria of the *Xanthomonas* genus that has attracted high interest due to its functional properties as a thickening or gelling hydrocolloid [168,169]. The primary structure of XG is composed of five repeating units, including a segment of two units of β-(1-4)-D-glucose, which are linked at positions one and four (similar to cellulose), and a trisaccharide side chain consisting of two mannose residues with a glucuronic acid residue between them is attached at C-3 at each second unit (Figure 9). The proximal mannose residue (which is close to the main chain) may be linked to an acetyl group at C-6, and the terminal mannose residue (distal to the main chain) may attach a pyruvate group between C-4 and C-6. The pyruvate and acetyl groups were not identified in all repeating units; their content is influenced by fermentation conditions. For example, high pyruvate content was induced by the presence of citric acid [170], and low pyruvate XG was obtained by reducing the amount of phosphate or magnesium in the growth media [171]. Furthermore, the fermentation conditions influence the acetyl to pyruvyl ratio on the outer mannose [172]. Thus, the primary structure of the xanthan chains and the type of substitution at the outer mannose unit (acetate or pyruvate) influence the stability of the xanthan conformation. It was shown that segments rich in units that are acetylated on the outer mannose stabilize the xanthan conformation [173].

**Figure 10:**
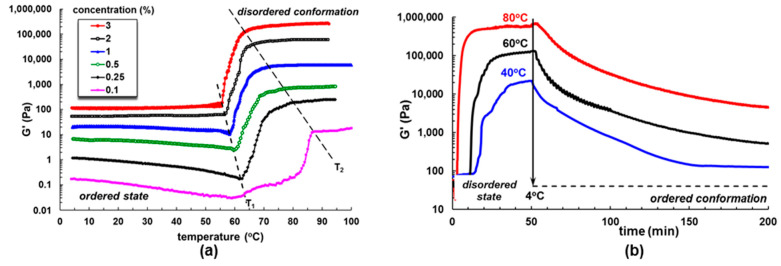
The elastic modulus for XG aqueous solutions: (**a**) different concentrations, at increasing temperature (heating rate of 1 °C/min); (**b**) concentration of 2%, denatured at different temperatures and then rapidly cooled to 4 °C. Adapted with permission from [175], copyright 2020, Elsevier.

Most of the practical applications of XG are related to peculiar rheological properties that are strongly influenced by primary and secondary structures. Depending on the temperature, ionic strength, pH, or shear rate, XG adopts in aqueous media either rigid helical ordered conformation or flexible conformation as disordered coil [64,65]. Ordered conformation presents the side chains folded and associated with the main backbone by hydrogen bonds (i.e., methyl group of acetate residues binds to hemiacetal oxygen atoms of alternate D-glucose residues from the main chain). Thus, the acetyl groups stabilize the ordered conformation [174]. The side chains are no longer associated in the disordered state as a result of the repulsion electrostatic interactions between COO− groups from pyruvate residues. Under favorable conditions, a coaxial antiparallel 5_1_ double helix is formed through an enthalpically-driven process [64]. Even after deacylation, XG chains are involved in intramolecular hydrogen bonding associations between alternate hydroxyl groups at C-3 and the adjacent hemiacetal oxygen atoms of the D-glucosyl residues. An increase of apparent viscosity and viscoelastic moduli during temperature rise from 25 °C to ~35 °C may be attributed to the breakdown of the two alternate intramolecular associations in the side chains. When the helix-to-coil transition is initiated, the overall dynamic is less influenced by the backbone. At higher temperatures (>45 °C), the disordered conformation is extended, and the chains interpenetrate forming entanglements with strong electrostatic and noncovalent interactions, which determine a sharp increase of rheological parameters [175,176,177]. Figure 10a presents the variation of the elastic modulus (G’) as a function of the temperature for XG aqueous solutions equilibrated at 4 °C and heated with a constant heating rate of 1 °C/min. For temperatures below T_1_, G’ slowly decreases by raising temperature, a tendency more pronounced for concentrations below 1%. Above T_1_, G’ sharply increases by several orders of magnitude, faster as the concentration of the solution becomes higher.

Above T_1_, XG undergoes a transition from ordered conformation to disordered state, and this process ends at a temperature T_2_, when the random coil conformation prevails. Higher concentrations (*c*) suppose more released macromolecules, and thus the disordered coils appear at a slightly lower temperature. It was observed that the difference ΔT = T_2_ − T_1_ scales as *c*^0.5^; also, around T_1_, the viscoelastic moduli G’ and G” scale as *c*^2.5^, and above T_2_ they vary as *c*^3^ [175].

After the disruption of XG structure either due to high shear or temperature, structure recovery is very slow and incomplete in aqueous solutions (Figure 10b) or in the presence of a low amount of salt. Recovery becomes faster and complete in high-salt environments [175,178]. A complete ordered conformation was obtained for NaCl concentrations higher than 0.01 M. The salt is recommended to be added into the XG solution prior to thermal treatment [168]. This shifts the transition temperature to higher values [175], ensuring the stability of xanthan-based formulations, such as suspensions or creams.

For a high pyruvated, single-stranded XG, no molecular increase occurred during the conformational change induced by salt addition, showing that this process takes place through intramolecular interactions [179]. The salt presence increases the transition temperature by reducing the effective charge of the xanthan chains. A linear relationship between the natural logarithm of the salt concentration and the reciprocal temperature was observed [180].

In aqueous solutions, XG displays high viscosity even at low concentrations [57], making this polysaccharide a valuable thickening or stabilizing agent of high interest in food, agriculture, drilling fluids (coil industry), paint, detergents, cosmetic and pharmaceutic products (creams, pastes), etc. [168,181,182].

However, in ordered state, XG is not able to undergo any chemical transformations [183] or to form networks using usual gelation methods [184]. Beyond a critical polymer or salt concentration, XG chains aggregate in disordered state determining an increase of viscoelastic parameters, as discussed above (Figure 10a) [175]. Yield stress behavior (liquid-like behavior only above a certain shear stress value) and rheopectic effects (an increase of the apparent viscosity before reaching a steady state) were observed for concentrated solutions of XG at 25 °C [181].

**Figure 11:**
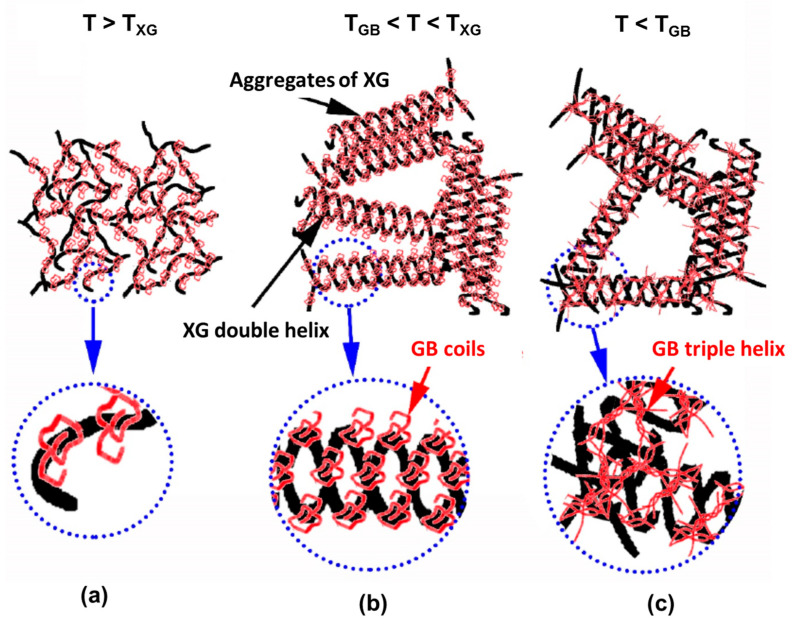
The gelation mechanism of GB/XG mixtures, based on their conformations as a function of temperature: (**a**) T > T_XG_; (**b**) T_GB_ < T < T_XG_; (**c**) T < T_GB_. Adapted with permission from [186], copyright 2017 Elsevier.

XG forms multifunctional hydrogels with other natural compounds, such as gelatin [185,186], CS [187], GG [188], collagen [189], and alginate [190]. A synergistic and complex gelation occurs when XG is mixed with gelatin B (GB) [186], both biomolecules with specific coil-to-helix transitions for temperatures T_XG_ above 60 °C and T_GB_ below 30 °C, respectively, slightly influenced by concentration. The complex gelation mechanism proposed by the authors is depicted in Figure 11. Near the isoelectric point of GB (pH range between 4.5 and 5.5) and above T_XG_, soluble GB/XG complexes are formed (Figure 11a), decreasing the charge density of XG. For temperatures between T_GB_ and T_XG_, the soluble complexes assemble into interpolymer complexes (Figure 11b), where XG presents an ordered structure. The interpolymer complexes form large associate structures stabilized by GB molecules. Temperatures below T_GB_ determine enhanced local GB concentration promoting the triple helix formation and gelation of GB. An increase of G’ over time suggested the formation of an elastic network consisting of mixtures of GB and XG helices (Figure 11c). These transitions (coil-to-helix and helix-to-coil) are thermoreversible [186].

3D porous bio-inks that mimic the physical and mechanical properties of soft tissue scaffolds were obtained from xanthan gum/nanocellulose [191], revealing viscoelastic properties that allow good control of printed structure composed of multiple layers with high resolution and shape fidelity. The addition of cellulose nanocrystals significantly enhanced the mechanical properties of composite hydrogels (the elastic and compressive moduli of a few kPa, similar to soft tissues). For bioprinting applications, a deep rheological investigation of bio-inks and analysis of the viscoelastic parameters are needed to ensure shape fidelity and high quality in correlation with cell viability of the 3D constructs [140].

### 2.5. Marine (Algal) Polysaccharides

#### 2.5.1. Alginate

**Figure 12:**
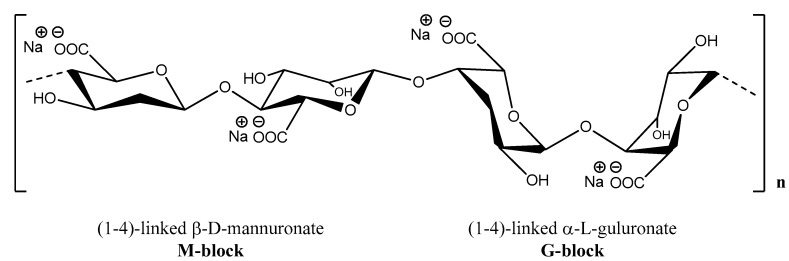
Chemical structure of sodium alginate.

Alginate (Alg) is a polysaccharide derived from brown algae constituted from long linear chains of block copolymers of (1,4)-linked β-D-mannuronate (M) and α-L-guluronate residues (G). Generally, the term of alginate denotes all alginic acid derivatives and their salts. Frequently, it is used as the salt derivative—sodium alginate (Figure 12)—that is water soluble and able to form stable solutions and gels, less sensitive to temperature as compared with other polysaccharides [192,193]. Depending on the source or extraction conditions, the molecular weight of Alg is usually in the range of 3.2 × 10^4^ to 4 × 10^5^ g/mol [194]. The Alg solutions behave as thixotropic and pseudoplastic fluids [30] regardless of the algal sources [192]. The M/G ratio and the number of repeating G-blocks depend on the Alg origin and influence network formation and gel characteristics [195,196,197].

The viscoelastic properties of Alg-based hydrogels can be tuned by varying the molecular weight, varying the length and distribution of M and G blocks or M/G ratio, selecting the appropriate crosslinking method (ionic or covalent), or using chemical modification of alginate chains [192,194,198,199]. The strength of Alg gels improves with increasing the molecular weight up to 2.4 × 10^5^ g/mol (corresponding to a value of intrinsic viscosity of 4.8 dL/g); above this limit the effect is negligible, regardless of Alg concentration [200].

Many pharmaceutical, biomedical, and food applications are based on the ability of Alg to undergo a sol–gel transition in the presence of divalent or multivalent cations, a process independent of temperature. Therefore, a three-dimensional network results, which is insoluble in water and thermally irreversible due to the increase of the rotation barrier around glycosidic bonding [192,201,202].

The M sequences from Alg are soft and elastic and determine a delay in the gelation process. Alg chains with high M content (M/G > 1) form elastic gels, while gels with low M content (M/G < 1) are compact, hard, and brittle [203,204,205]. An ultrasound treatment applied to Alg chains increases hydrophobic interactions and interfacial activity. At the same time, the M/G ratio is diminished, determining an increase of chain stiffness [143].

Ion-induced Alg gelation occurs mainly by electrostatic interactions between the–COO^−^ groups and divalent or multivalent cations (Ba^2+^, Cu^2+^, Sr^2+^, Fe^2+^, Zn^2+^, Mn^2+^, Al^3+^, Fe^3+^, etc.), with the formation of polyelectrolyte complexes [194,206]. The gel strength is influenced by the ionic radius of the cations (as for example Ba^2+^ and Sr^2+^ form a stronger gel as compared with Ca^2+^ ions) [207]. The most studied ion-induced gelation is crossinking Alg macromolecules in the presence of Ca^2+^. The intermolecular junctions are formed by chelation of Ca^2+^ between G-blocks by a bimolecular mechanism (the gel strength increases with the square of Alg concentration) [200] through the so-called “egg-box” association [7,194,208,209,210,211]. Strong interactions of cations with COO^−^ groups of guluronic acid from different chains occur in the cavities formed by pairing up of the successive G sequences forming two-fold structures that create cavities to hold Ca^2+^ in the binding sites [7,194,210,212]. Ca^2+^ cations coordinate with six oxygens (O2, O3, O6) from two neighbouring G units and with one to three oxygens of H_2_O and form a stable “egg-box” structure [210,213]. Further, the G blocks adopt helix conformations and contribute to the formation of polysaccharide networks [194,209,210,214]. Various studies have discussed three successive steps of G-block gelation in the presence of of Ca^2+^ ions governed by electrostatic interactions, according to the “egg-box” model [209,215,216,217,218,219]. These include the interaction of Ca^2+^ with a guluronate unit resulting in monocomplexes; the pairing of monocomplexes into “egg-box“ dimers; and the lateral association of the “egg-box“ dimers into multicomplexes. The Alg chains are zipped by simultaneous intracluster associations of “egg-box“ dimers and intercluster separation. The excess of Ca^2+^ neutralizes the negative charges of Alg, hindering the association between “egg-box“ dimers [209].

When alginate and divalent cations are mixed directly, gels form almost instantly, and it is difficult to assess the gelation kinetic; in addition, the structure of the network is heterogeneous. To overcome this difficulty, rheometers with modified geometries were adopted for the experimental investigations. In situ real-time gelation kinetics of Alg were monitored by using a custom-made rheometer geometry (a custom-made lower plate combined with an upper commercial cone) that allowed injecting CaCl_2_ into the sample during the rheological measurements [220]. A continuous increase of viscoelastic moduli and stiffness of hydrogels was observed by increasing alginate concentration up to 3 wt.%. For a given Alg concentration, a balance between the supplied volume and the available Ca^2+^ cations was suggested. For example, keeping constant the sample volume (0.15 mL) and alginate concentration (2 wt.%), the hydrogel stiffness could be tuned through the concentration of the crosslinker (G′ increased by a factor of seven when CaCl_2_ concentration was increased from 37.5 mM to 150 mM). A dimensionless parameter, i.e., the ratio [mole Ca^2+^]/[mole COO^−^], was defined and considered proportional to the fraction of intermolecular crosslinks [221]. This parameter was used for monitoring in situ gelation of Alg using rheological measurements. Different procedures for Alg gelation were applied, assuming either internal or external mechanisms [222], that determined different distributions of crosslinking points and thus different properties of the gels. The internal gelation procedures used inactive forms of Ca^2+^ (such as insoluble salts) as crosslinkers, prolonging the initiation stage of gelation [32,222,223]. The mechanical properties of these gels (network strength and elasticity) were improved by the addition of glucose as a cosolute (up to 30%) [224]. The external crosslinking method supposed the direct exposure of Alg chains to active forms of Ca^2+^ [222,224], leading to greater matrix strength, stiffness, and permeability than with internally crosslinked gels. This last method is preferred for many applications, such as coating or drug encapsulation [222]. Spherical shaped microparticles as pH-responsive drug carriers for therapeutic applications were obtained by internal gelation of Alg, but their application is not appropriate for longer release times, when further reducing of the surface porosity or hydrophilicity of the microspheres are necessary [223].

In situ gelation was monitored by using filter paper impregnated with CaCl_2_ solution on a Petri dish placed on the lower plane of the rheometer. Alg solution was loaded onto a semipermeable membrane positioned on the filter paper, and the gelation was monitored through the viscoelastic parameters [225]. When gelation was completed, a new filter paper impregnated with ethylenediamine tetraacetic acid or sodium citrate allowed determination of the gel stiffness in the presence of calcium chelators.

By controlling the characteristics of Alg chains and obtaining methods, a wide range of mechanical properties are accessible using Alg-based hydrogels. Generally, the shear modulus ranges from 0.02 to 40 kPa, and the compression modulus of alginate gels was found in the range of less than 1 kPa to more than 10^3^ kPa [226]. The nanoporous network, which is formed by ionic crosslinking, is inert to cells and provides an appropriate mechanical microenvironment for cells [198], drug delivery matrices for therapeutic agents, and applications in wound dressing and 3D bioprinting [227]. In addition, among the divalent cations, calcium is the most suitable for high-absorbent alginate hydrogels used in various environmental and biomedical applications [7].

Alginate hydrocolloids are used as thickeners, emulsifiers, stabilizers, or pharmaceutical additives for foods, beverages, or biomaterials for medical and pharmaceutical industries [192]. The chelation, gelation, and hydrophilic properties of alginates have increased the interest for their use in food, cosmetics, and biomedical applications [210,227]. Alginate-based hydrogels with tunable properties are suitable platforms for biomaterials used in regenerative medicine or as bioresponsive drug delivery systems. They present structural similarities to ECMs, being frequently used for 2D or 3D cell cultures. As with the ECM model in cell biology or bioengineering studies, these versatile structures allow a better understanding of fundamental cell processes: interactions between cells and ECMs (elasticity or stiffness), cell–ECM adhesion, spreading, growth, proliferation, migration, differentiation, organoid formation, etc. ECM viscoelasticity can regulate these processes [228,229,230]. The elastic modulus for natural tissues varies from hundreds of Pascals in brain or fat tissue up to tens of GPa in bone [230]. Viscoelasticity can be evaluated through rheological studies by examining stress relaxation in response to deformation, creep in response to loading, or the ratio between dissipation to accumulation energy (quantified by the loss tangent, tanδ, in dynamic oscillatory tests). Most ECMs and tissues submitted to a deformation display substantial stress relaxation over a timescale of 10–10^4^ s and tanδ (as the ratio between the viscous, G”, and elastic, G’, moduli) value around 0.1 [229,230]. The stress relaxation can be controlled by mixing different ratios of high- and low-molecular-weight alginate samples, or by coupling with short poly(ethylene glycol) chains [193].

Hydrogels obtained from Alg with molecular weights of 2.8 × 10^4^ g/mol (fast relaxing), 7 × 10^4^ g/mol (medium relaxing), and 2.8 × 10^5^ g/mol (slow relaxing); different arginine–glycine–aspartate ligand densities (between 1.5 × 10^−7^ M and 1.5 × 10^−3^ M); and calcium crosslinking densities (from 8 mM to 33 mM) presented the values of initial elastic modulus from 3 kPa to 20 kPa. The values of τ_1/2_ (the time required for the stress to drop to half of its initial value) obtained for stress relaxation in compression and shear tests were about 10^3^ s in slow relaxing gels, 180 s in medium relaxing gels, and 70 s in fast relaxing gels. The loss tangent value decreased from approx. 0.08, registered for fast relaxing gels, to about 0.06, obtained for slow relaxing networks [229].

The deposition of type II collagen and aggrecan in slow relaxing Alg-based hydrogels was limited to the region adjacent to the cells [193]. A more interconnected cartilage matrix with a higher area of type II collagen and aggrecan deposition was obtained in the faster relaxing gels. Displacements were evidenced in fast relaxing hydrogels, which are opaquer, whereas no movements were observed in slow relaxing hydrogels. Faster stress relaxation in the hydrogels promotes the formation of wider areas of cartilage matrix and fabrication of more extended interconnected cartilage matrices [193].

By conjugating peptides (Arg-Gly-Asp) to Alg, cell adhesion and spreading were facilitated [198]. Cell adhesion on surfaces is an important concept in the field of tissue engineering due to the improvement of cell proliferation properties [231]. RGD is a protein-derived peptide motif with cell adhesion capacity found in natural ECM elements, such as fibronectin, collagen, and tenascin C. The tripeptide sequence is the most effective ligand for integrin-mediated cell adhesion. The biological process involves a cascade of four overlapped reactions, which are cell attachment, spreading, actin-skeleton formation, and focal-adhesion formation [231,232]. The reactions are important for transmitting signals related to cell behavior and the cell cycle [232]. An RGD cell-recognition motif has been attached or blended with natural materials, such as gelatin, alginate, chitosan, cellulose, keratin, and elastin, or with synthetic materials, such as polyvinyl alcohol and polyacrylamide, in order to enhance cell adhesion and provide better biocompatibility [233,234].

Using physical and/or chemical crosslinking, Alg provides versatile structures with a wide applicability range, from scaffolds for tissue engineering, wound dressings, and vehicles for bioactive agents (drugs, proteins, and antimicrobial or antioxidant agents) to sensors and actuators [192,235,236,237,238].

**Figure 13:**
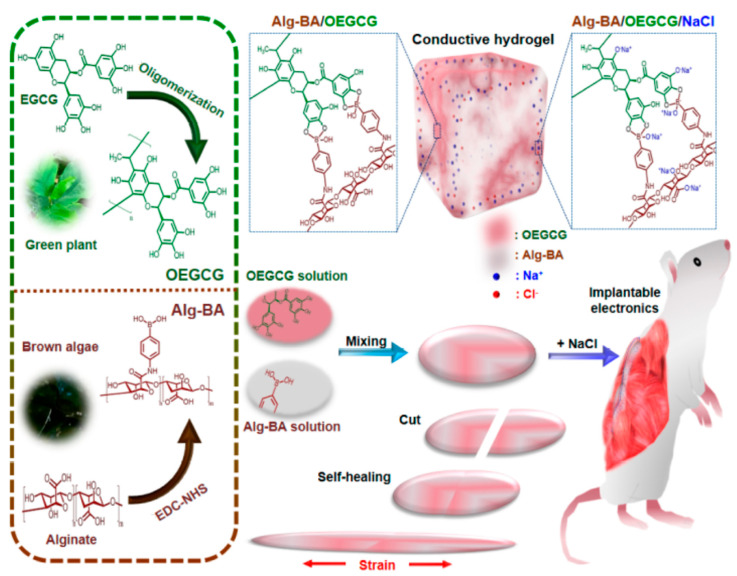
Schematic presentation of Alg-BA/OEGCG hydrogel preparation and their potential for implantable electronics. Alg-BA = boronic-acid-modified alginate; OEGCG - oligomerized epigallocatechin; NaCl - sodium chloride; EDC-NHS (1-(3-dimethylaminopropyl)-3-ethylcarbodiimide hydrochloride/N-hydroxysuccinimide) [239].

Conductive, self-healing, and stretchable hydrogels with dynamic covalent bonds were obtained from boronic-acid-modified alginate (Alg-BA) and oligomerized epigallocatechin gallate (OEGCG) for implantable electronics (Figure 13) [239].

Cotton gauze was coated with Alg, glycerol (as plastifiant), and tannic acid (antimicrobial and antioxidant agent) for wound dressing applications [240]. Glycerol addition destroys the intermolecular hydrogen bonds between Alg chains and determines a continuous decrease of shear viscosity with increasing the glycerol concentration. This material showed cell viability and antibacterial activity (>95% viable colony reduction) against *S. aureus* and *E. coli*.

Alg is used frequently for 3D bioprinting due to its biological and tunable rheological properties. In the presence of Ca^2+^, low concentrations of Alg form weak networks able to maintain cell viability during bioprinting [241]. An amidic alginate derivative crosslinked with 1,3-diaminopropane forms hydrogels with stiffness and viscoelasticity suitable for nucleus pulposus replacement (G’ > 10 kPa) [242]. Viscoelastic moduli (G’ and G”) of hydrogels were monitored over time to evidence structural changes by loading different shear stress values corresponding to daily activities. The results have shown behavior comparable with non-degenerated human nucleus pulposus. The viscoelastic moduli (G’ and G”) presented closed values before and after the dynamic stress tests. The dehydration of hydrogels during these tests was of max. 15% [242]. The potential of Alg hydrogels for spinal cord injury treatment was discussed in a recent review [243].

The use of single-component Alg hydrogels presents some limitations due to poor mechanical properties, limited biodegradation, or dissolution due to releasing divalent ions. Some inconveniences were improved with Alg oxidation [244] or by preparing new materials using Alg in combination with other natural polymers, proteins, or peptides [245,246,247,248,249].

By incorporating a small amount of cellulose or oxidized cellulose nanofibers into the alginate/gelatin hydrogels, their printability (shear thinning and thixotropic behavior) and mechanical properties (tensile and compressive stress) were improved [247,250].

#### 2.5.2. Carrageenans

Carrageenans (Carr) denote a group of sulfated linear polysaccharides (extracted from red algae) that present in their structure alternating units of D-galactose and 3,6-anhydro-galactose linked by α-1,3 and β-1,4-glycosidic linkage. There are different types of carrageenans that differ by their degree of substitution on their free hydroxyl groups and the position of sulphate groups within the structural units. Other carbohydrate residues that may be found in the Carr structure are glucose, xylose, and uronic acids, and as substituents methyl ethers and pyruvate groups can be also present [251,252]. Thus, the following forms have been identified: Kappa (κ)-, Iota (ι)-, Lambda (λ)-, Mu (μ)-, Nu (ν)-, and Theta (θ)-Carr. The most investigated and used forms are the gels of κ-Carr and ι-Carr. Furthermore, λ-Carr is used as thickening agent. Due to their rheological (thickening/gelling) and biological (antiviral, immunomodulatory, antitumoral, and anticoagulant) properties, these carrageenans are used not only in food and pharmaceutical industries, but also in cosmetics and tissue engineering [252].

**Figure 14:**
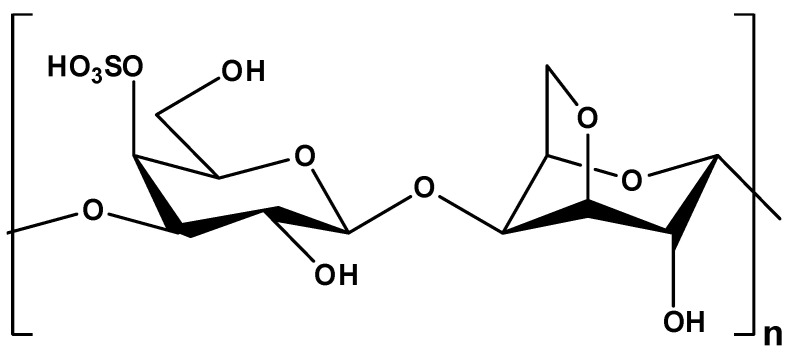
Chemical structure of κ-carrageenan.

**Figure 15:**
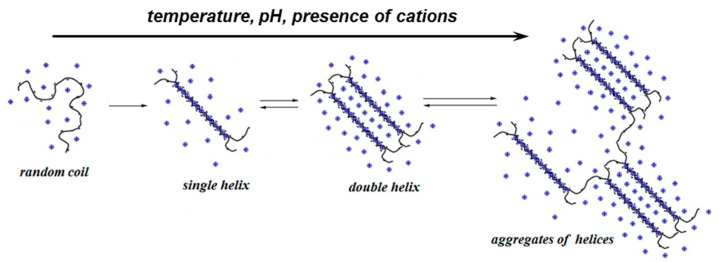
Schematic presentation of the reversible changes in the conformation of κ-Carr.

κ-Carr (Figure 14), a copolymer of (1→3) linked β-D-galactose-4-sulfate and (1→4) linked 3,6-anhydro-α-D-galactose, with about 25% (*w*/*w*) sulphate and 34% (*w*/*w*) 3,6-anhydro-D-galactose [252], is able to undergo reversible gelation induced by temperature, pH, or presence of cations, and it is used in the food industry as a gelling or thickening agent. The network includes single or double helices (Figure 15) with the sulphate groups towards their external side [253]. In the presence of cations, the network strength is considerably improved due to conformational ordering aggregation [54,254].

**Figure 16:**
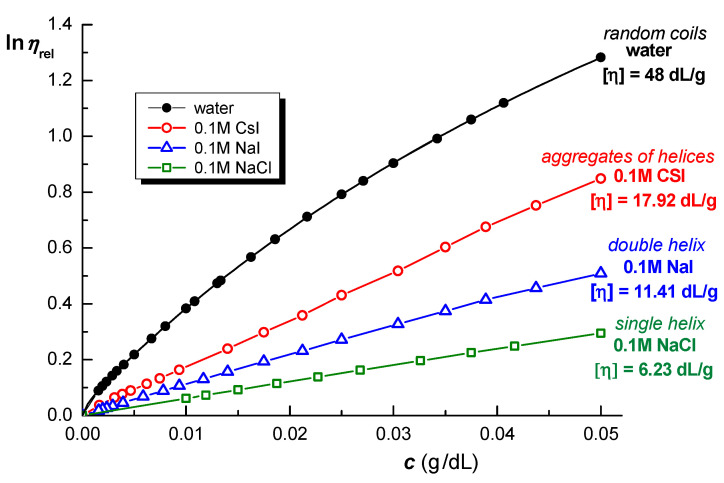
Viscosity of dilute solutions of κ-Carr in aqueous solutions and in presence of 0.1 M monovalent salts. Adapted with permission from [54], copyright 2019, Elsevier.

A specific behavior of κ-Carr chains is the ability to form thermoreversible gels in the presence of electrolytes; the effect is more pronounced for alkaline metal counterions of high atomic number [66]. The intrinsic viscosity, [η], of κ-Carr in aqueous solutions (random coils) was found to be 48 dL/g [54]. In 0.1 M solutions of monovalent salts, the values differ considerably. In the presence of NaCl, the single helix is formed and [η] = 6.23 dL/g, whereas in NaI a double helix conformation is favored and [η] = 11.41 dL/g (dissimilar counter-ion, I^−^, viscosity approx. double as compared with Cl^−^). The shielding effect is less pronounced in the presence of I^−^, and the double helix is stabilized by the presence of I^−^ in the solvent. In CsI solution, there is dissimilar cation and counter-ion and [η] = 17.92 dL/g (three times higher value), suggesting the formation of associates consisting of aggregates of helices (Figure 16) [54]. Anions and cations influence the behavior of κ-Carr in a different manner, and counter-ions are more efficient as compared with co-ions in inducing coil-to-helix transitions. In the presence of the same cation, Na^+^, the effect of I^−^ is more pronounced as compared with Cl^−^ one, and I^−^ causes an increased stiffness in the ordered conformation of κ-Carr [67].

It was shown that in 0.1 M aqueous solutions of mixed salts (NaI and CsI), κ-Carr helices associate into superhelical rigid rods in a certain domain of salt composition (above a critical mole fraction of Cs, *f*_Cs_ > 0.4) [255]. In the presence of NaI, the double helices formed by the adjacent spiral chains contain sulfate groups oriented towards their external parts [251]. The change of the conformational-associative properties of κ-Carr was executed with progressive modifications of the ionic environment. Thus, the stepwise association of κ-Carr helices was followed in NaI solutions by adding an increasing amount of CSI [54]. Under specific conditions of polymer and salt concentration, the helices aggregate and lead to gelation [256]. Gels with low values of viscoelastic moduli were also obtained above 0.9% (*w*/*w*) κ-Carr in presence of NaI, although this salt is known to impede the gelation of κ-carr. It was shown that κ-Carr gelation occurs in two different ways [257]: coil-to-helix-to-gel or coil-to-helix-to-superhelical-rod-to-gel. The first way is a reversible association of helical dimers in solutions of NaI or in NaI and low content of CsI. The second type of gelation supposes the formation of superhelical rods, and the values of the viscoleastic moduli increase [258].

The cations, such as K^+^, Rb^+^, Cs^+^, and NH_4_^+^ stabilize the helix and stimulate gel formation. In the coil state, the presence of Na^+^, Li^+^, H^+^, or (CH_3_)_4_N^+^ delays or cancels the network formation [66]. The disordered state is reached by increasing the temperature and by cooling the solution at room temperature until the coil-to-helix transition occurs. At constant ionic strength, the gelling temperature is found in the following order: Rb^+^ > Cs^+^ > K^+^ > NH_4_^+^ ≥ (CH_3_)_4_N^+^ > Na^+^ > Li^+^ [67], which corresponds with the ability of cations to enhance gelation [173] and only shows slight variation for bivalent counterions: Ba^2+^ > Ca^2+^ > Sr^2+^ > Mg^2+^ > Zn^2+^ > Co^2+^ [259]. Ca^2+^ binds between the double helices (rather than the single ones) and confers high thermal stability to κ-Carr. The dimerization is similar to that occurring for Alg—the so-called “egg-box” structure with a single array of cations between two chains. Ca^2+^ can be replaced with monovalent cations: K^+^ acts indirectly by suppressing charge, and the thermal stability is lower as compared with Ca^2+^. The effect of Na^+^ addition is different; it decreases the thermal stability of the helix–helix aggregates and lowers the order–disorder transition point [260]. The cation concentration required to induce the coil-to-helix transition increases in the order: Rb^+^ < Cs^+^ ≈ K^+^ < Na^+^ < Li^+^ [253], whereas the resulting gel strength is in the opposite order [261]. Thus, it was supposed that divalent cations determine direct crosslinking between κ-Carr helices, and monovalent cations bind only single sulphate groups along κ-Carr chains. There were more junction zones in divalent-cation-invoked gels. Ca^2+^ in the presence of K^+^ showed a synergistic effect in mechanical properties [253].

### 2.6. Fungal Polysaccharides

#### Pullulan

**Figure 17:**
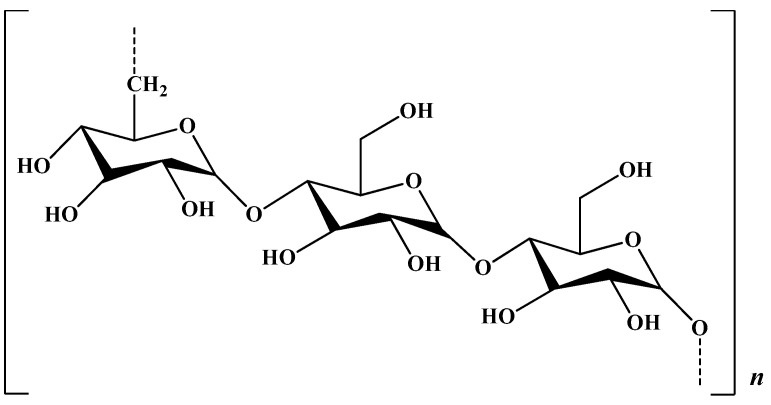
Chemical structure of pullulan.

Pullulan (PULL), an exopolysaccharide produced by certain fungi, such as *Aureobasidium pullulans*, presents in its structure maltotriose units connected through α-(1→4) glycosidic bonds, the consecutive maltotriose units being linked to each other by α-(1→6) glycosidic bonds (Figure 17).

Due to its unique properties, such as chain flexibility, solubility in water, high adhesion to biological surfaces, ability to form transparent, thin edible films, biocompatibility, and biodegradability, it is extensively used as a food ingredient, in packaging, in cosmetics or dermatocosmetics, as a pharmaceutical excipient for protein stabilization, in biomedical applications as hydrogels for drug delivery systems, for wound healing, for tissue engineering applications, and for wastewater remediation [262,263,264,265,266,267,268].

In physiological conditions, PULL and bovine serum albumin (BSA) form complex structures mediated by Na^+^ cations. Due to favorable intermolecular interactions, more than one macromolecule of BSA is incorporated into the PULL coil resulting a mixed coil that increases the viscosity and the osmotic pressure of biological fluids, two important physiological and clinical parameters [269].

In combination with other biomolecules, PULL is incorporated into wound dressings, inducing antimicrobial, antioxidant, and nonimmunogenic properties. For example, synergistic hyaluronic acid-grafted pullulan succinate and chitosan physical networks present antibacterial activity and accelerate skin wound repair [270]. In addition, the clinical potential of biocompatible porous pullulan/dextran hydrogels for mice survival after liver failure was recently demonstrated [271].

The oxidation of PULL is a way to convert its neutral macromolecules into polyelectrolytes with valuable properties that allow expansion of application ranges. However, such chemical reactions can also lead to severe chain degradation depending on the reaction conditions and polysaccharide structure [272,273,274,275].

PULL and its derivatives present improved properties and are suitable for drug delivery systems [263,267,276], wound dressings, or tissue engineering applications [277,278,279,280,281]. A glutathione and pH dual-responsive drug delivery system composed of CM and oxidized PULL hydrogel was used to encapsulate methotrexate (an anticancer drug) [282,283] loaded on mesoporous silica nanoparticles [282].

## 3. Proteins

**Scheme 2:**
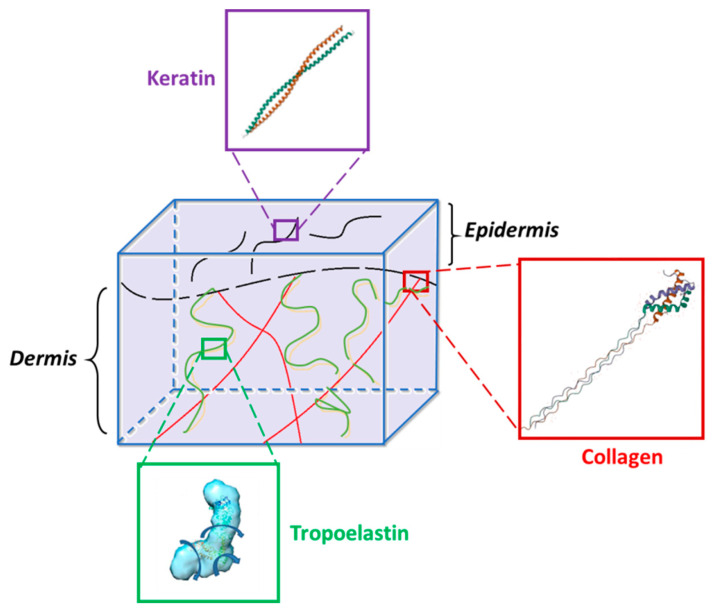
Illustrative representation of proteins from ECM: collagen [286], keratin [287], tropoelastin [288]. Structure of tropoelastin was adapted with permission from [288], copyright 2020, Elsevier.

Proteins, the building blocks of life, are well known for their diverse functions. Their main advantage is that primary structure might predict function, supramolecular assembly, and proper protein folding [284]. Hydrogels based on natural proteins have received a lot of attention in the past two decades [285]. Besides well-studied collagen hydrogels, keratin, elastin, and silk hydrogels have been increasingly investigated. These smart biomaterials show a high potential in a vast range of applications, from food to cosmetic and pharmaceutical industries. Collagen, keratin, and elastin are the main components in the natural ECMs (Scheme 2), and these proteins are used in most tissue engineering applications.

### 3.1. Collagen

Collagen (Coll) is the most abundant protein in nature, and it plays both functional and structural roles, such as promoting cell migration and proliferation, an important component of connective tissue, ligaments, tendons, and blood vessels [289]. There are at least 29 known forms of Coll classified according to their functions and homology. Type I is the most studied and widespread collagen form. This type comprises about 90% of the protein in human connective tissue, mainly responsible for bone development and organ support [285].

**Figure 18:**
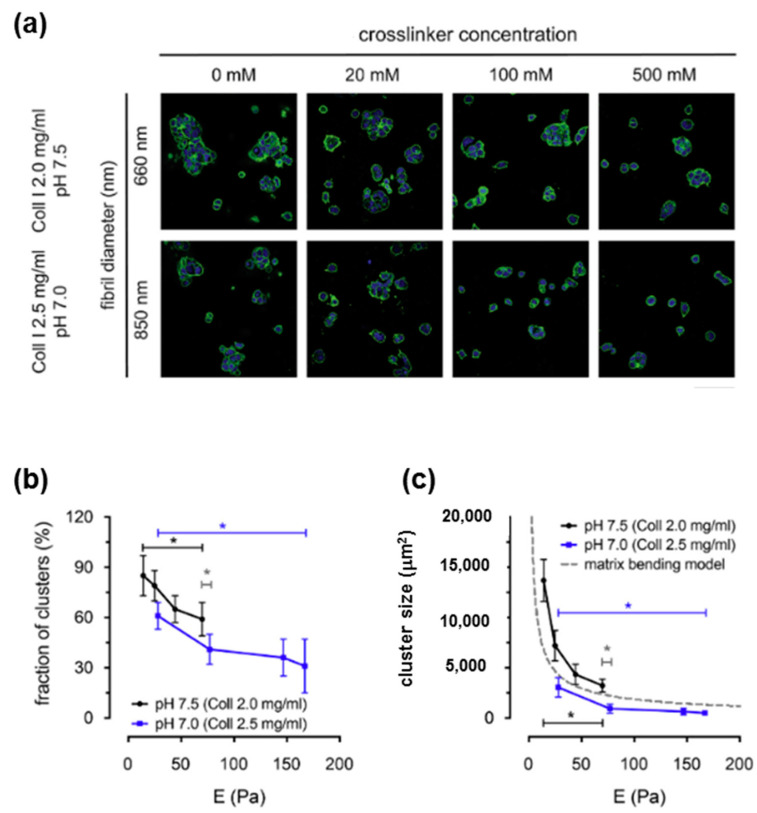
Correlation between Coll I fibril bending stiffness and cell clustering of MCF-7 cells. (**a**) The confocal laser scanning microscopy images of MCF-7 breast cancer cells cultivated in 3D Coll I matrices with fibril diameters of 660 nm and 850 nm and with postmodification by different concentrations of the crosslinker (from 0 to 500 mM) after 5 days of cultivation. Images show nuclei (blue) and actin filaments (green). (Scale bar: 100 μm). (**b**,**c**) Quantitative analysis of cell clustering of MCF-7 cells. Cells without contact to other cells were assigned as single cells. Fraction of clusters (ratio of number of cell clusters and single cells) (**b**) and cluster size (**c**) were plotted as a function of matrix elastic modulus, E. Dashed line in (**c**) depicts the simple model of cluster formation limited by matrix bending energy (gray dashed line). Data are shown as mean ± SD; *—significance level of *p* < 0.05. Adapted with permission from [290], copyright 2019, Elsevier.

For cancerous tissues, the mechanical (elasticity and viscosity) and structural (microstructure and Coll density) characteristics of ECM modulate the behavior of cancerous breast cells [290,291]. In mammary tumors, an increase of Coll fibril diameter and a crosslinking mediated by lysyl-oxidase take place, determining a higher tissue stiffness as compared with the normal one [292]. Investigations have shown that cluster formation in tumor cells is regulated by the fibril bending stiffness controlled by intrafibrilar crosslinking [290]. Figure 18 shows the cluster formation for a tumor model (breast cancer cell line MCF-7) as a function of the elastic modulus. An increase of the fibril diameter or intrafibrillar crosslinking of Coll I, using 1-ethyl-3-(3-dimethyl aminopropyl)-carbodiimide as a crosslinker, reduced the cluster occurrence of MCF-7 cells (Figure 18a). A nonlinear decrease in the fraction of clusters (Figure 18b) and average cluster size (Figure 18c) occurs when the elastic modulus matrix increases (Figure 18b,c). Thus, these results evidenced the correlation between cancer cell phenotypes and the mechanical properties of the Coll fibrils [290].

Mechanical strength of Coll materials has been reported as insufficient as compared to the native Coll, due to the lack of crosslinking [293]. Hybrid hydrogels were created by fusing collagen with other (macro)molecules in order to enhance their mechanical properties. A variety of studies have evidenced a synergy between Coll and HA in the conception of hydrogels with enhanced mechanical properties for biomedical purposes.

A recent study describes a convenient method for obtaining an injectable hydrogel suitable for cartilage regeneration by rapid thiol/maleimide chemical reaction and thiol oxidation reaction as the primary and secondary self-crosslinking networks. Subsequently, isolated chondrocytes were appended into the HA hydrogel for cartilage expansion and type I collagen (Coll I) was supplementarily added for mechanical strength improvement [294]. Addressing a similar topic, another group of scientists managed to prepare bone marrow mesenchymal stem-cells-laden HA-Col I hydrogel using an enzymatic crosslinking for tissue engineering applications. Briefly, the Coll I and HA precursor solutions were mixed, and the crosslinked process was initiated by adding horseradish peroxidase and hydrogen peroxide as a substrate. Hydrogel strength and formation rate depend on the substrate concentration (a higher concentration of H_2_O_2_ leads to fast formation of the hydrogel). The study reported good biocompatibility and physico-chemical properties of the prepared hydrogel. Additionally, in vivo exploratory research revealed that hyalin cartilage repair could be possible [295]. Hyaluronate/Coll hydrogels can also be prepared in situ for sutureless corneal defect repair. The procedure involves a bio-orthogonal strain-promoted azide-alkyne [3+2] cycloaddition reaction between HA-azide and dibenzocyclootyne-Coll intermediate solutions. The main advantage of this type of hydrogel formation is that it can be made under ambient conditions, without chemical catalysts and initiators, heat, or light. In addition, the obtained hydrogel is highly transparent and displays comparable optical and physical properties with natural corneal stroma [296].

Coll/CS hybrid crosslinked hydrogels can overcome the weak points of mono-biomolecular hydrogels in terms of degradation and mechanical properties. In a recent study, a Coll/CM hydrogel was prepared via 1-ethyl-3-[3-dimethylaminopropyl] carbodiimide hydrochloride (EDC) and N-hydroxysuccinimide (NHS) as chemical crosslinkers. This is a low-cost and easy method to obtain new amide bonds between the reactants. The hydrogel synthesis was performed by mixing the Coll/CM substrate solutions with EDC/NHS as coupling agents. Further, RGD peptide was additionally included into the mixture in order to enhance the scaffold’s biocompatibility and adhesiveness. These gels were recommended for cartilage regeneration [297]. Crosslinked Coll can absorb high amounts of water and can act as a self-standing electrolyte membrane in an aqueous electrolyte used for electrochemical devices [298].

### 3.2. Keratin

Keratin is the major protein of hair, nails, wool, hooves, and horns. This protein has a high resistance to common proteases, such as pepsin or trypsin, and is rich in cysteine (Cys), proline (Pro), glycine (Gly), and serine (Ser). Furthermore, keratin is insoluble in water, weak acids, organic solvents, and alkaline solutions [299]. The stability and stiffness of this natural protein are based on its high content of Cys residues (7–20%), which can easily form intra- and interchain disulfide bonds. These bonds can be physically or chemically dislocated to create water-soluble keratin extracts. The ability of proteins to dissolve in water is crucial when creating materials for biomedical applications. For example, wound dressings are one of the most appreciated applications for keratin-based materials [300]. According to a recent study, an innovative wound dressing hydrogel-loaded with vitamin C was conceived based on keratin, XG, and gelatin. Firstly, the keratin/XG/gelatin hydrogel was obtained using glycerol as a crosslinking agent. Initially, for the adsorption process, the hydrogel was submerged into a vitamin C solution for 24 h. Afterward, the loaded material was analyzed by in vitro hydrogel degradation and used for release studies, cytotoxicity studies, and Coll staining procedures. It is known that vitamin C is involved in post-translational modification of collagen in fibroblasts. As the study concluded, the vitamin C released from the hydrogel increased the Coll maturation process in the cells [301]. A wound dressing with antibacterial properties based on the combination between CMC and keratin from human hair was provided by another study. The main function of the dressing was to deliver topical antibiotic clindamycin [302,303], which is effective against burn as well as skin and tissue infections caused by *S. aureus*. The hydrogel was tested for in vitro clindamycin release, antibacterial activity, antibiotic impact on fibroblast attachment, and viability. Overall, the results showed good antibacterial activity, more than 90% cellular viability, and proliferation [302].

A research team developed novel techniques for manufacturing an injectable keratin hydrogel for hemostasis and drug delivery in wound healing applications. The crosslinking strategy was based on a dynamic exchange reaction between Au(I)-thiolates (Au-S) and the keratin’s disulfide bonds (S-S) under physiological pH conditions. The advantage of this method is the harnessing of the existing thiol groups in keratin, without the need for any structural modification. Moreover, the results of rheological tests, in vivo hemostatic evaluation, and wound healing tests demonstrated a promising hemostatic and efficient wound healing effect [304]. Among wound healing and hemostatic applications, keratin hydrogels were also used in human hair disorders. For this cosmetic purpose, the keratin-based hydrogels were filled with halloysite nanotubes [305,306], which are nanofillers for biopolymeric matrices. As reported in the literature, halloysite–protein composites are used in several applications, such as sustained release of bioactive molecules, dye removal, and enzyme immobilization. Thus, these active principles are recommended to be incorporated into haircare formulations [305].

### 3.3. Elastin

Elastin is an essential protein responsible for the resilience and elasticity of native ECM and organs [285,307]. Moreover, it is predominant in tissues that require extension and relaxation repeatedly, such as lungs, blood vessels, ligaments, and skin [308]. The precursor of elastin is tropoelastin, which is a soluble monomer secreted by elastogenic cells. Tropoelastin crosslinking is achieved using enzymatic catalysis to form insoluble mature protein [309].

Due to its resilience, elastin can form hydrogels with high porosity and high levels of cell penetration [310]. Elastin-based materials have applications mainly in wound healing and regenerative medicine [311]. In most studies, elastin is typically incorporated into hybrid hydrogels, along with other molecules, such as polysaccharides, proteins, or peptides, to shape the properties of the final material. For example, a recent study described the fabrication of a plasma–elastin hybrid hydrogel for skin regeneration. The material was created to overcome the shortcomings of plasma-derived fibrin hydrogels, which exhibit low mechanical properties, rapid contraction, and long-term degradation. Therefore, a dual crosslinked network based on plasma-fibrin alternated with different elastin-like recombinamers was created. These findings reveal the effective generation of novel networks with enhanced mechanical properties [312]. In order to enhance the cellular adhesion and biomimicking functions of Alg hydrogels, a group of scientists studied the interactions of three proteins, including elastin, with graphene oxide (GO) platelets. In this experimental work, the adsorption of elastin, type I Col, and BSA on the GO surface was followed. Then, the protein-GO particles were incorporated into the alginate hydrogels and analyzed through in vitro cell viability tests. The results indicated that the elastin and BSA-GO hydrogels improved cell viability and the release of a therapeutic protein [313].

Another review shows the association between elastin and silk to create silk-elastin-like protein (SELP)-based hydrogels for drug and gene delivery. The study presented a variety of controlled releasing profiles for bioactive agents from loaded SELP hydrogel matrices, as well as possible liquid embolic applications [314].

Elastin-like polypeptides show the desired properties, similar to those of elastin, that are the focus of researchers in the most recent related studies. Further along in this paper, the interesting aspects of this special category of polypeptides will be discussed.

## 4. Peptides

Peptides are unique building blocks for supramolecular assembly that can be designed from up to 20 amino acids. Their self-assembly capacity is dictated by the peptide’s sequence (primary structure), which can adopt α-helix or β-sheet secondary structures [315]. The main disadvantages of peptides are susceptibility to enzymatic degradation, fast kidney clearance, and possible immunogenic-induced responses [316]. Attaching peptides to either natural or synthetic polymers can solve some of these issues.

### 4.1. Elastin-like Polypeptides

As previously discussed, elastin-like polypeptides (ELPs) have similar elastic properties to native elastin, but improved solubility. This particular category of recombinant polypeptides is composed of a repeated amino acid sequence (VPGXG)*_n_*, where *X* comprises any amino acid, except proline, a fact that disrupts the folding behavior, and *n* represents the number of pentapeptide sequences [317,318]. By varying the guest residue (X), the length of the polypeptide, and its concentration, ELPs are able to reversibly undergo a phase separation around the lower critical solution temperature (LCST) or above the transition temperature. These unique properties enable ELPs to be used as drug delivery vehicles, protein reservoirs, or bioactive compound carriers. Additionally, according to numerous studies, ELPs can be produced recombinantly with enzyme-cleavable sites, adjustable aggregation temperatures, and cell-adhesive moieties without generating any immune response [318]. This is a very promising aspect in cancer therapy and for designing novel anticancer delivery systems. For example, doxorubicin or paclitaxel was conjugated to the ELPs and used for targeted delivery to the tumor site [319].

Some authors have already pointed out the association between ELPs and hyaluronic acid in order to develop hybrid hydrogels with versatile applications, especially in the biomedical field [320]. In this context, few studies highlighted the preparation of ELP/HA hydrogels. One of them introduces a hydrogel with antimicrobial, elastic, and adhesive properties comprising methacrylated hyaluronic acid (MeHA)-ELPs for tissue engineering applications. A photo-crosslinked method was used to generate the MeHA-ELP hybrid hydrogel. Zinc oxide (ZnO) nanoparticles were added into the hydrogel precursor solution in order to provide antimicrobial characteristics. The obtained material was further investigated using in vitro cytocompatibility and adhesive and antibacterial properties, as well as using in vivo biodegradation and biocompatibility studies. According to the authors, the hydrogel might promote cell proliferation and be integrated into the host tissues, without any risk of triggering inflammatory responses [321]. On the same topic, a hydrogel of ELP-Col incorporating recombinant human bone morphogenetic protein-2 (rhBMP-2) and doxycycline to induce osteogenesis has been reported in the literature. This study followed doxycycline and rhBMP-2 release tests, mechanical studies, cell morphology, and viability. The hydrogel showed activity against *E. coli*, *S. sanguinis*, and *P. aeruginosa*, as well as good cell attachment, proliferation, and differentiation, and is a promising bone regeneration material [322].

### 4.2. Collagen-like Peptides

**Figure 19:**
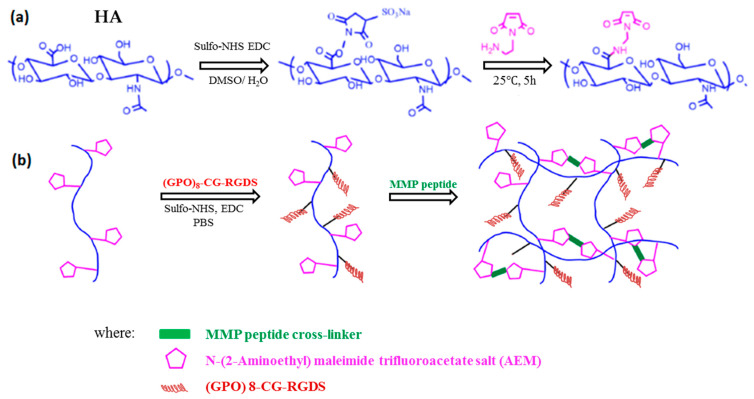
Synthesis of collagen-mimetic peptide-HA hydrogel. (**a**) Functionalization of HA with N-(2-aminoethyl) maleimide trifluoroacetate salt (AEM): (**b**) Synthesis of HA-collagen-mimetic peptide hydrogel. With permission from [324], copyright, 2020 Elsevier.

Short peptides designed with the repetitive collagen motif (Gly-X-Y)n, where X and Y stand for proline (Pro or P) and hydroxyproline (Hpy or O) residues, are referred to as collagen-like peptides (CLPs) or collagen-mimetic peptides (CMPs). The motif confers the triple helical conformation that the native protein possesses on the synthetic structure [323]. CLPs present better solubility, immunogenicity, and thermal stability compared to native collagen, as well as similar biological activity [324,325]. CLP/polysaccharide hydrogels are mainly used in regenerative medicine as biomaterials for bone repair, cartilage regeneration, wound healing dressings, or targeted drug delivery [326]. For example, a recent study managed to obtain a biomimetic hydrogel system based on a CMP structure linked to a hyaluronic acid backbone to promote the differentiation from BMSCs into chondrocytes for cartilage-grow applications [324]. Firstly, HA was functionalized with maleimide groups (HA-MAL) and analyzed using ^1^H-NMR. Further, the (GPO)_8_-CG-RGDS collagen-mimetic peptide was conjugated to the previous functionalized polymer. This peptide contained a (Gly-Xaa-Yaa)n sequence and could mimic the structure and biological activity of natural collagen. The molar ratio of the reaction between the CMP and crosslinker was 3:1. The preparation of the hydrogel was achieved using incubation of the HA-MAL-CMP and matrix metalloproteinase (MMP) at 37 °C for more than 30 min. Figure 19 illustrates the synthetic route to design the biomimetic hybrid hydrogel.

**Figure 20:**
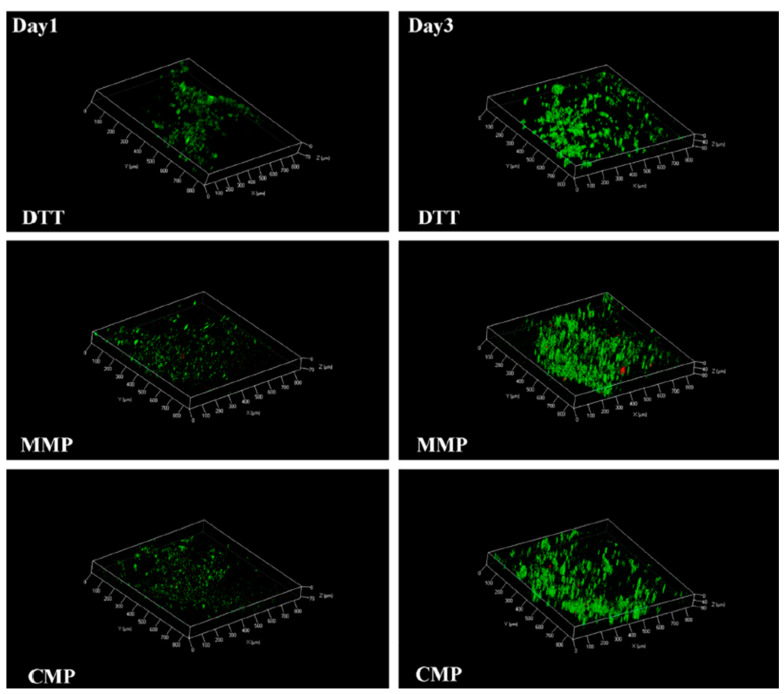
Cell distribution inside the hydrogels at days 1 and 3. With permission from [324], copyright 2020, Elsevier.

Cell viability was followed at 1 and 3 days after culturing the cells entrapped in the gels. Figure 20 shows the cell distribution compared on DTT (DL-1,4-Dithiothreitol used as stabilizer), MMP, and CMP hydrogels from day one and day three. After day one, the DTT media showed significantly lower viability than that of the other two groups. The cell viability on the CMP hydrogel was the strongest after three days, while the DTT group remained the weakest. The results showed the potential of the collagen biomimetic-peptide-hydrogel to influence differentiation of the BMSCs into cartilage [324].

On a slightly different topic, a recombinant collagen-peptide, RHC, was conjugated with CS to obtain a chemically crosslinked hydrogel with better biological and mechanical properties for wound healing applications. Briefly, both RHC and CS were solubilized in distilled water. RHC was mixed with EDC binding reagent to activate the carboxyl groups. The activated peptide was transferred into the chitosan solution and incubated at 37 °C. The structure of the obtained hydrogel was characterized by FTIR and ^1^H-NMR and further analyzed using cytotoxicity of gel extraction, cell encapsulation, and histological and immunofluorescence studies. The study reported superior conjugation efficiency of hydrogels, biocompatibility, and excellent potential as burn injury treatment [327].

### 4.3. Antimicrobial and Antioxidant Peptides

Short peptide sequences, with between 2–40 residues of amino acids, that exhibit a variety of biological activities are called bioactive peptides. According to their mechanism of action, bioactive peptides are classified as antimicrobial agents, antioxidants, antihypertensive agents, metal chelators, etc. [328,329]. As an alternative to specific drugs, bioactive peptides exhibit fewer side effects while combating oxidative stress and microbial infections. Moreover, these compounds could have natural origins and could be assimilated from different beans, seeds, plants, or animal sources.

#### 4.3.1. Antimicrobial Peptides

One of the most significant events in medicine was the discovery of antibiotics, which are currently employed extensively in a variety of industries including food and agriculture. However, the new generation of antibiotic-resistant microorganisms represents one of the main global public health concerns [330]. Therefore, new mechanisms of action or targets are explored in order to combat drug-resistant infections, in which antimicrobial peptides (AMPs) represent promising therapeutic options [331]. AMPs are produced as a defense mechanism by all living organisms and regulate the immune system against invasive pathogens [332]. In fact, gramicidin, the first AMP, was isolated from *B. brevis* and demonstrated in vivo and in vitro antibacterial activity against a range of Gram-positive bacteria [333].

AMPs are briefly classified into two groups based on their cell-targeting activity: intracellular targeting groups and extracellular targeting groups. The intracellular targeting mechanism involves peptides with transmembrane transport capacity. Peptides are able to form pores in the cell membrane and translocate into the cell. Further, AMPs inhibit the processes of replication, translation, and transcription by binding to nucleic acids and disturb the energy metabolism and cell cycle by affecting some enzymatic systems. The extracellular mechanism includes cell wall targeting. AMPs inhibit the synthesis of peptidoglycans, the main component of the bacterial cell wall, by selectively binding to lipid II, the precursor molecule of peptidoglycans. Eventually, the cell wall is destroyed, and the bacteria can no longer perform their function [330,334].

The antimicrobial activity and specificity of AMPs are modulated by physico-chemical parameters of the peptides and membranes of the microorganisms. The bacteriostatic and bactericidal effects depend on amino acid composition, more precisely the charge of the peptide, secondary structure, hydrophobicity, and amphipathicity. Most of the AMPs carry a net positive charge that enables electrostatic interactions with the negatively charged membranes, which are the first step in binding to the bacterial cell wall. Hydrophobicity is an essential feature because it allows the peptide to penetrate the lipid bilayers of the bacterial cell membrane. Many studies have demonstrated that antimicrobial activity increases at an optimal percentage of hydrophobic residues. Once connection is formed between the peptide and microbial cell, AMPs may adopt various secondary structures, such as α-helical, β-sheet, and extended or mixed α/β-structures. The secondary structure of the peptide influences the antimicrobial mechanism and selective cytotoxicity. The conformational flexibility of antimicrobial peptides is attributed to their amphipathic nature, which activates both hydrophobic and hydrophilic domains within the peptide [335,336].

#### 4.3.2. Antioxidant Peptides

Antioxidant activity of peptides is related to structural characteristics, such as amino acid composition, sequence, molecular mass, and hydrophobicity. According to a similar review, antioxidant peptides consist of three to six amino acid residues with a range of molecular weights from 400 to 650 g/mol. An additional finding of the study reveals that 70% of the 42 identified antioxidant peptides have molecular weights below 10^3^ g/mol. Peptides with high antioxidant capacity consist of hydrophobic amino acids, which are considered key factors in scavenging radicals. His, Pro, Gly, Val, and Lys are among the most frequently tested hydrophobic amino acids. However, other studies have mentioned the ability of Trp, Tyr, and Phe, the three aromatic amino acids, to significantly improve antioxidant capacity [337,338]. The antioxidant properties are determined mostly by in vitro ability to scavenge free radicals (hydroxyl, 2,2-diphenyl-1-picrylhydrazyl, superoxide), metal chelation, reducing ferric iron to ferrous, and inhibiting lipid peroxidation [339]. 1,1-Diphenyl-2-picrylhydrazyl (DPPH) assay is one of the most popular spectrophotometric methods for antioxidant capacity determination, which involves a direct reaction between the radicals and antioxidants. DPPH is a stable nitrogen radical with a deep blue color. The delocalization of electrons gives rise to a deep violet color, featuring an adsorption band at around 517 nm. The DPPH radical is reduced in the presence of antioxidant molecules, which donate their hydrogen atoms. As an outcome, the solution’s color changes to a pale yellow, pointing to a reduced form [340,341].

Oxidative stress has become one of the main problems of the modern, unhealthy lifestyle, being involved in the development of several chronic diseases, such as atherosclerosis, diabetes, and cancer [342,343,344,345,346,347]. Accumulation of reactive oxygen species (ROS) activates the pro-inflammatory and pro-apoptotic pathways by triggering uncontrolled reactions with proteins, lipids, and DNA [347]. Both synthetic and natural antioxidants manifest significant ROS scavenging abilities in order to maintain human health. Nevertheless, synthetic antioxidants such as butylated hydroxytoluene (BHT) and butylated hydroxyanisole (BHA) may present carcinogenic side effects. Therefore, it is encouraging to consume and use natural antioxidant compounds as food preservatives rather than synthetic antioxidants [348,349]. Plants, animals, dairy, and marine products represent inexhaustible resources for natural biopeptides [347].

Plant-based foods provide nutrients, such as sugars, proteins, lipids, and biologically active substances [348,349]. For example, mung bean peptides exhibit antioxidant, antitumor, and antidiabetic properties [350]. Peptides extracted from walnut residues have also been explored in recent years due to their antioxidant and antihypertensive properties [351,352]. Other examples of plant sources from which peptides are derived are cottonseeds, potatoes, and spinach, while peptides isolated from seaweed and aquatic animals are part of marine and animal sources [353,354,355,356].

### 4.4. Nucleopeptides

Nucleobase-bearing peptides, also known as nucleopeptides, are hybrid molecules where the nucleic acid base is attached to a peptide skeleton via its 1- or 9-position. Their structural moieties endow the occurrence of many intermolecular interactions between adjacent nucleopeptide chains or short nucleopeptides and polynucleotides [357]. A recent study has shown that autocatalytic and cross-catalytic processes involving short nucleopeptide replicators are jointly controlled by the nucleobase motifs’ hybridization and the peptide segments’ propensity for assembly. Within networks of complementary nucleopeptides, unequal assembly-dependent replication triggers a selectivity toward the emergence of a particular species. This type of assembly process most probably was further translated to biological assemblies such as cellular organelles and viruses [358].

Small size nucleotide-aromatic dipeptides (Thymine-FF and Thymine YY) were characterized for their self-assembly properties using UV spectroscopy, circular dichroism (CD), and dynamic light scattering (DLS). Although these peptides tend to form aggregates with diameters higher than 300 nm in solution, they are not able to interact significantly with homoadenine DNA and RNA as revealed by CD spectroscopy [359].

Two hydrogelators, one containing thymine, a phenylalanine moiety and a glycoside, and the second two aromatic moieties flanked in a similar fashion, were also reported. These supramolecular structures displayed biocompatibility to HeLa cells and good resistance to proteases. Fluorescence microscopy studies support nucleopeptide–nucleic interaction and their entry into nuclei [360,361]. Conversely, when a saccharide moiety was placed at the N-terminal of FRGD or naphthyl-AFRGD peptide and the nucleobase at C-terminal, these conjugates exhibited excellent cell biocompatibility but were unable to self-assemble in an aqueous environment [362].

Two nucleotide-tetrapeptides (guanosine-GKFF-OH and guanosine-GKFF-NH_2_) were used as a template for self-assembly investigation. TEM images, in the absence or presence of KCl (10 equivalents), support the idea that slight structural changes on peptides’ C-terminus yield distinct morphologies. For the amidated construct, a G-ribbon architecture was preferred [363]. A novel thymine-decorated artificial hexameric peptide based on L-diaminopropanoic acid was also studied for its capacity to interact with single- and double-stranded DNA and RNA and later for its capacity to be used in antiviral strategies [364]. Another report focused on three thymine-containing dodecamers that were synthesized and their ability to hybridize with dA12. The homopolymer of thymine-containing diaminobutiric did not bind to oligodeoxynucleotide, and its monomer incorporation into thymine-containing N-(2-aminoethyl)glycine dodecamers at the N-terminus and in the middle of the main chain led to weak binding to the targeted DNA as compared to reference dodecamer [365]. Chiral nucleopeptides (as heteropolymers) were obtained using γ-diaminobutiric and lysine thymine amino acids as starting precursors. The binding of hexamer (one monomer consists of a pair of different thymine amino acids) to dA12 and polyA was revealed using CD and UV spectroscopies [366]. Nucleic acid binding ability of a dithymine tetra-L-Serine nucleopeptide was also reported. The binding behavior was demonstrated using CD and in silico studies. This nucleopeptide might induce a better structuration when interacting with its random deoxyoligonucleotide partner [367]. A trinucleo-heptapeptide was also synthesized and was characterized by its capacity to interact with single-stranded oligonucleotides. The self-assembly capacity of a nucleopeptide, more precisely, Nap-FFK(t)GK(c)GK(t), where t is thymine and c is cytosine, was superior in the presence of single-stranded DNA (ssDNAs). This new compound also displayed the ability to interact with plasmid DNA and might be used to deliver hairpin DNA into cells with the possibility of affecting their behavior and function [368]. Two sets of homo-thymine nucleopeptides were synthesized as stated by another elegant study. Among these two 12-mer nucleopeptides, which comprise a thymine-functionalized L-ornitine and an adjacent L-2,4-diaminobutyric acid, respectively, as an arginine unit, were able to bind to specific DNA or RNA targets and to cross cellular and nuclear membranes [369]. Additionally, the interaction of nucleopeptides with RNA molecules is greatly influenced by backbone stereochemistry. As a result, stable complexes with RNA are being generated by nucleopeptides that only contain alternating L-arginine and L-Lysine residues and a thymine nucleobase [370].

A recent study investigated the gelation of thymine-triphenylalanine triggered by Good’s buffer and salts. The gels displayed long-term stability in cell culture conditions and short-term cell viability and proliferation as compared to widely used materials [371]. A library of sixteen nucleo-tripeptides, each having two phenylalanine residues at C-terminus, was constructed in order to identify potential candidates that form hydrogels in physiological conditions. The nanofibrous hydrogel’s formation was dictated using base pairing and aromatic π–π stacking interactions. Proliferation of 3T3 fibroblasts in a media containing 0.075–0.6 % (wt.) nucleo-triphenylalanines was also confirmed, and thus, this type of compound is suitable for biomedical applications [372]. A biomimetic G-quadruplex hydrogel was prepared using bioconjugation of cytosine-functionalized dipeptide with guanosine and 4-formylphenylboronic acid. This injectable hydrogel exhibited antibacterial activity, and its biocompatibility was demonstrated on HEK293T and MCF-7 cell lines [373].

Supramolecular assemblies of nucleopeptides (particularly in a N-terminal thymine-capped noanapeptide, Thymine-FFKKFKLKL) could selectively supply ATP to cancer cells, enhancing the effectiveness of the drug doxorubicin. Thus, these nucleopeptides are suitable as gelators, which serve as valuable entities for identifying cellular bioactive molecules [374].

The scientific community has taken advantage of the ability of nucleopeptides and pseudopeptides to form well-ordered architectures. These supramolecular tools having spherical, worm-like, or cylindrical structures have applications in biomedicine due to their biocompatibility and biodegradability. In a recent review, the characteristics of these supramolecular systems are succinctly outlined with an emphasis on their most essential applications [375]. Consequently, gene release towards the target cell could be accomplished using these supramolecular assembled scaffolds [361].

A recent review describes various nucleopeptide-based hydrogels. Briefly, this paper has aimed to cover nucleopeptides having incorporated nucleotide, nucleoside derivatives, or peptide nucleic acid segments. In addition, low-weight hydrogel-forming peptides having a covalently linked nucleobase are also mentioned. The physico-chemical and mechanical properties, their characterization by various techniques, and their biological applications are also summarized [376]. Another reported study reveals nucleodipeptides that have a nucleobase moiety attached at C-terminal via a triazole or an amide linker. The morphology of self-assembled N-capped-nucleopeptides (with protected groups such as Boc, tBu, and Cbz) was revealed using high-resolution surface investigation techniques. Nucleodipeptides based on triazoles have the capacity to form nanospheres with exciting potential in the field of bioelectronics [377]. In addition, a novel N-terminal acetylated nucleopeptide, having 18 amino acids and a nucleotide repeat unit (GC) inside the peptide main chain, was reported. This unusual nucleopeptide was acting as a template for ferric oxide selective mineralization as an oriented nanotape [378].

Carba-nucleopeptides were also obtained via photocatalysis using Rhodamine B. The reaction mechanism of dehydroalanine peptide with a bromonucleobase was elucidated using experimental and computational approaches [379].

## 5. Conclusions and Future Outlook

**Scheme 3:**
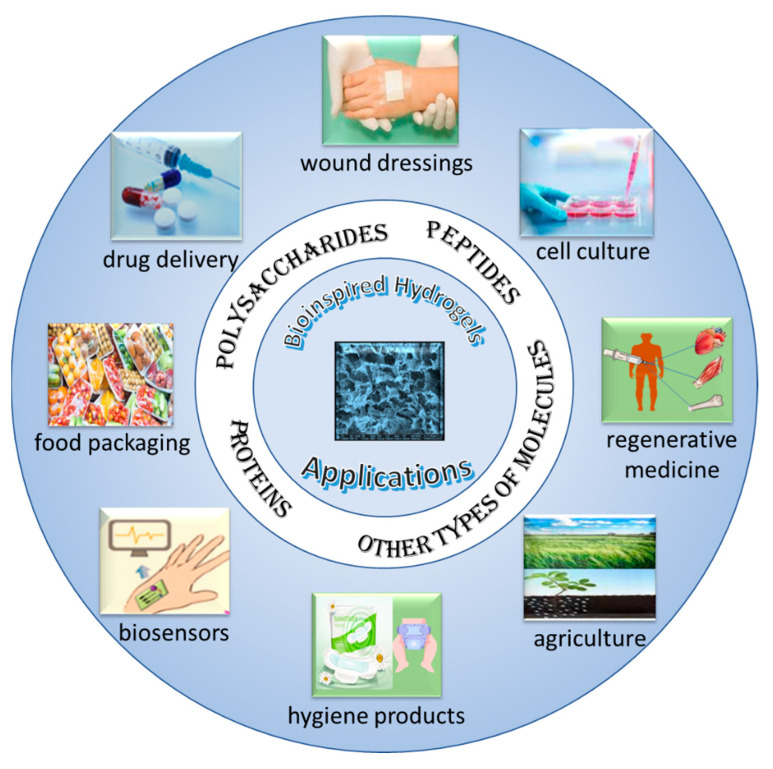
A brief view of the most important biomedical and environmental applications of bioinspired hydrogels.

A high diversity of hydrogels is designed from biomolecules with complex structures and architectures at different scales. These networks are continuously studied, and their performance is improved in order to reduce synthetic materials and replace such materials with bio-based ones to the highest possible extent. Natural resources are is inexhaustible, and the main advantages of biomolecules are related to their ability to confer valuable biological and functional properties onto hydrogels, such as biocompatibility, antimicrobial properties, biodegradability, and nontoxicity. Thus, this research area is of great interest, and many efforts are coming together to improve the functionalities of existing biomaterials as much as possible, in order to meet the increasing requirements related to biomedical and environmental applications as well as increasing the quality of life. Without ending the discussion, Scheme 3 presents potential applications of bioinspired hydrogels.

Huge creative efforts are converging towards new materials as reflected in an avalanche of papers published in recent years, with some innovative hydrogels presenting a high potential for practical applications. The bioinspired hydrogels are suitable for manufacturing hygiene products, biosensors, bioimaging, foods and food packaging, matrices for drug transport and delivery, wound dressings, cell culture, DNA- or RNA-targeted delivery, regenerative medicine, 3D printing of biological tissues, water depollution, agriculture, bioelectronics, etc.

This review evidences some attractive and unique properties of various biomolecules that recommend them for preparing new customized hydrogels or for improving existing ones for a specific application. Some valuable characteristics of bioinspired hydrogels are: biocompatibility; biodegradability; hemostatic, antioxidant, antibacterial and anti-inflammatory activities; self-healing ability; stimuli responsiveness; electrical conductivity; etc. The range of applications is extended either by using physical methods for preparing various multicomponent and multifunctional hydrogels, or by chemical modification and convenient crosslinking of biomolecules.

Long-term storage and distribution of biomolecules, without losing their valuable characteristics or losing easy access to bioresources, also needs to be taken into account. In vivo tests represent a determinant step for hydrogel applications. Interdisciplinary studies for hydrogel design, characterization, and in vitro investigations [55,92,116,189,380,381], along with access to preclinical and clinical studies to ensure safety and efficacy of new materials [98,271,382,383,384,385], will accelerate the application of new concepts and revolutionary materials. Last but not least, education regarding the protection of nature, which generously offers us a variety of biomolecules, must be promoted beyond the academic framework.

## Data Availability

Data are available on request.

## References

[1] Bashir S., Hina M., Iqbal J., Rajpar A.H., Mujtaba M.A., Alghamdi N.A., Wageh S., Ramesh K., Ramesh S. (2020). Fundamental concepts of hydrogels: Synthesis, properties, and their applications. Polymers.

[2] Yang Q., Peng J., Xiao H., Xu X., Qian Z. (2022). Polysaccharide hydrogels: Functionalization, construction and served as scaffolds for tissue engineering. Carbohydr. Polym..

[3] Huang H., Dong Z., Ren X., Jia B., Li G., Zhou S., Zhao X., Wang W. (2023). High-strength hydrogels: Fabrication, reinforcement mechanisms, and applications. Nano Res..

[4] Liu S., Tang J., Ji F., Lin W., Chen S. (2022). Recent advances in zwitterionic hydrogels: Preparation, property, and biomedical application. Gels.

[5] Chao Y., Klein J. (2022). Lipids and lipid mixtures in boundary layers: From hydration lubrication to osteoarthritis. Curr. Opin. Colloid Interface Sci..

[6] Yang J., Wang S. (2023). Polysaccharide-based multifunctional hydrogel bio-adhesives forwound healing: A review. Gels.

[7] Makarova A.O., Derkach S.R., Khair T., Kazantseva M.A., Zuev Y.F., Zueva O.S. (2023). Ion-induced polysaccharide gelation: Peculiarities of alginate egg-box association with different divalent cations. Polymers.

[8] Zhang H., Shi L.W.W., Zhou J. (2023). Recent developments of polysaccharide-based double-network hydrogels. J. Polym. Sci..

[9] Srivastava N., Richa, Choudhury A.R. (2021). Recent advances in composite hydrogels prepared solely from polysaccharides. Colloids Surf. B.

[10] Teodorescu M., Bercea M., Morariu S. (2018). Biomaterials of poly(vinyl alcohol) and natural polymers. Polym. Rev..

[11] Bercea M., Gradinaru L.M., Morariu S., Plugariu I.A., Gradinaru R. (2022). Tailoring the properties of PVA/HPC/BSA hydrogels for wound dressing applications. React. Funct. Polym..

[12] Bercea M. (2022). Bioinspired hydrogels as platforms for life-science applications: Challenges and opportunities. Polymers.

[13] Bercea M. (2022). Self-healing behavior of polymer/protein hybrid hydrogels. Polymers.

[14] Li M., He X., Zhao R., Shi Q., Nian Y., Hu B. (2022). Hydrogels as promising carriers for the delivery of food bioactive ingredients. Front. Nutr..

[15] Raina N., Pahwa R., Thakur V.K., Gupta M. (2022). Polysaccharide-based hydrogels: New insights and futuristic prospects in wound healing. Int. J. Biol. Macromol..

[16] Dumitriu S. (2004). Polysaccharides, Structural Diversity and Functional Versatility.

[17] Díaz-Montes E. (2022). Polysaccharides: Sources, characteristics, properties, and their application in biodegradable films. Polysaccharides.

[18] Smith P.J., Ortiz-Soto M.E., Roth C., Barnes W.J., Seibel J., Urbanowicz B.R., Pfrengle F. (2020). Enzymatic synthesis of artificial polysaccharides. ACS Sustain. Chem. Eng..

[19] Nichifor M. (2023). Role of hydrophobic associations in self-healing hydrogels based on amphiphilic polysaccharides. Polymers.

[20] Jitaru S.C., Neamtu A., Drochioiu G., Darie-Ion L., Stoica I., Petre B.A., Gradinaru V.R. (2023). A diphenylalanine based pentapeptide with fibrillating self-assembling properties. Pharmaceutics.

[21] Gradinaru L.M., Bercea M., Lupu A., Gradinaru V.R. (2023). Development of polyurethane/peptide-based carriers with self-healing properties. Polymers.

[22] Reek R. (2017). The three-dimensional structures of amyloids. Cold Spring Harb. Perspect. Biol..

[23] Woolfson D.N. (2023). Understanding a protein fold: The physics, chemistry, and biology of α-helical coiled coils. J. Biol. Chem..

[24] Zhang Z., Wang J., Xia W., Cao D., Wang X., Kuang Y., Luo Y., Yuan C., Lu J., Liu X. (2022). Application of hydrogels as carrier in tumor therapy: A review. Chem. Asian J..

[25] Gomez-Florit M., Pardo A., Domingues R.M.A., Graça A.L., Babo P.S., Reis R.L., Gomes M.E. (2020). Natural-based hydrogels for tissue engineering applications. Molecules.

[26] Callmann C.E., Thompson M.P., Gianneschi N.C. (2020). Poly(peptide): Synthesis, structure, and function of peptide−polymer amphiphiles and protein-like polymers. Acc. Chem. Res..

[27] Fontana C., Widmalm G. (2023). Primary structure of glycans by NMR Spectroscopy. Chem. Rev..

[28] Grasdalen H., Larsen B., Smidsrød O. (1981). ^13^C-NMR studies of monomeric composition and sequence in alginate. Carbohydr. Res..

[29] Wang J.Q., Nie S.P. (2019). Application of atomic force microscopy in microscopic analysis of polysaccharide. Trends Food Sci. Technol..

[30] Williams M.A.K., Marshall A., Haverkamp R.G., Draget K.I. (2008). Stretching single polysaccharide molecules using AFM: A potential method for the investigation of the intermolecular uronate distribution of alginate?. Food Hydrocoll..

[31] Meng Y., Shi X.D., Cai L.Q., Zhang S.H., Ding K., Nie S.P., Luo C.F., Xu X.J., Zhang L.N. (2018). Triple-helix conformation of a polysaccharide determined with light scattering, AFM, and molecular dynamics simulation. Macromolecules.

[32] Funami T., Noda S., Nakauma M., Ishihara S., Takahashi R., Al-Assaf S., Ikeda S., Nishinari K., Phillips G.O. (2009). Molecular structures of gellan gum imaged with atomic force microscopy (AFM) in relation to the rheological behavior in aqueous systems in the presence of sodium chloride. Food Hydrocoll..

[33] Moge B., Schindler B., Yeni O., Compagnon I. (2023). Fucose migration pathways identified using infrared spectroscopy. Angew. Chem..

[34] Paul S., Danilov A., Gokus T., Barth A. (2023). A novel infrared spectroscopic approach to study the interaction of amyloid-B with cell-penetrating peptides—A possible new class of drugs to combat Alzheimer’s disease. Biophys. J..

[35] Salas E., Gorfer M., Bandian D., Kaiser C., Wanek W. (2023). A rapid and sensitive assay to quantify amino sugars, neutral sugars and uronic acid necromass biomarkers using pre-column derivatization, ultra-high-performance liquid chromatography and high-resolution mass spectrometry. Soil Biol. Biochem..

[36] Eckelt J., Sugaya R., Wolf B.A. (2008). Pullulan and dextran: Uncommon composition dependent Flory–Huggins interaction parameters of their aqueous solutions. Biomacromolecules.

[37] Bercea M., Nichifor M., Eckelt J., Wolf B.A. (2011). Dextran-based polycations: Thermodynamic interaction with water as compared with unsubsituted dextran, 2. Flory-Huggins interaction parameter. Macromol. Chem. Phys..

[38] Eckelt J., Richardt D., Schuster K.C., Wolf B.A. (2010). Thermodynamic interactions of natural and of man-made cellulose fibers with water. Cellulose.

[39] Lu G., Crihfield C.L., Gattu S., Veltri L.M., Holland L.A. (2018). Capillary electrophoresis separations of glycans. Chem. Rev..

[40] Ruddick A., Goodall D.M. (1998). Use of capillary electrophoresis for conformation and size of biopolymer 1. Theory and application to amylose and dextran. Macromolecules.

[41] Toppazzini M., Coslovi A., Paoletti S. (2011). Capillary electrophoresis applied to polysaccharide characterization. Capillary Electrophoresis of Carbohydrates.

[42] Grabarics M., Lettow M., Kirschbaum C., Greis K., Manz C., Pagel K. (2022). Mass spectrometry-based techniques to elucidate the sugar code. Chem. Rev..

[43] Zaia J. (2004). Mass spectrometry of oligosaccharides. Mass Spectrom. Rev..

[44] Veillon L., Huang Y., Peng W., Dong X., Cho B.G., Mechref Y. (2017). Characterization of isomeric glycan structures by LCMS/MS. Electrophoresis.

[45] Imberty A., Pérez S. (2000). Structure, conformation, and dynamics of bioactive oligosaccharides: Theoretical approaches and experimental validations. Chem. Rev..

[46] Perez S., Makshakova O. (2022). Multifaceted computational modeling in glycoscience. Chem. Rev..

[47] Gray C.J., Migas L.G., Barran P.E., Pagel K., Seeberger P.H., Eyers C.E., Boons G.J., Pohl N.L.B., Compagnon I., Widmalm G. (2019). Advancing solutions to the carbohydrate sequencing challenge. J. Am. Chem. Soc..

[48] Tudu M., Samanta A. (2023). Natural polysaccharides: Chemical properties and application in pharmaceutical formulations. Eur. Polym. J..

[49] Ghiorghita C.-A., Dinu M.V., Lazar M.M., Dragan E.S. (2022). Polysaccharide-based composite hydrogels as sustainable materials for removal of pollutants from wastewater. Molecules.

[50] Burton B.A., Brant D.A. (1983). Comparative flexibility, extension, and conformation of some simple polysaccharide chains. Biopolymers.

[51] Stokke B.T., Smidsrød O., Brant D.A. (1993). Predicted influence of monomer sequence distribution and acetylation on the extension of naturally occurring alginates. Carbohydr. Polym..

[52] Dentini M., Rinaldi G., Risica D., Barbetta A., Skjåk-Bræk G. (2005). Comparative studies on solution characteristics of mannuronan epimerized by C-5 epimerases. Carbohydr. Polym..

[53] Smidsrød O., Haug A. (1971). Estimation of the relative stiffness of the molecular chain in polyelectrolytes from measurements of viscosity at different ionic strengths. Biopolymers.

[54] Bercea M., Wolf B.A. (2019). Associative behaviour of kappa-carrageenan in aqueous solutions and its modification by different monovalent salts as reflected by viscometric parameters. Int. J. Biol. Macromol..

[55] Vold I.M.N., Kristiansen K.A., Christensen B.E. (2006). A Study of the chain stiffness and extension of alginates, in vitro epimerized alginates, and periodate-oxidized alginates using size-exclusion chromatography combined with light scattering and viscosity detectors. Biomacromolecules.

[56] Banerjee A., De R., Da B. (2022). Hydrodynamic and conformational characterization of aqueous sodium alginate solutions with varying salinity. Carbohydr. Polym..

[57] Brunchi C.E., Morariu S., Bercea M. (2014). Intrinsic viscosity and conformational parameters of xanthan in aqueous solutions: Salt addition effect. Colloids Surf. B.

[58] Morariu S., Brunchi C.E., Bercea M. (2012). The behaviour of chitosan in solvents with different ionic strengths. Ind. Eng. Chem. Res..

[59] Tinland B., Rinaudo M. (1989). Dependence of the stiffness of the xanthan chain on the external salt concentration. Macromolecules.

[60] Spatareanu A., Bercea M., Budtova T., Harabagiu V., Sacarescu L., Coseri S. (2014). Synthesis, characterization and solution behaviour of oxidized pullulan. Carbohydr. Polym..

[61] Buliga G.S., Brant D.A. (1987). Temperature and molecular weight dependence of the unperturbed dimensions of aqueous pullulan. Int. J. Biol. Macromol..

[62] Ioan C.E., Aberle T., Burchard W. (2000). Structure properties of dextran. 2. Dilute solution. Macromolecules.

[63] Smidsrød O., Glover R.M., Whittington S.G. (1973). Relative extension of alginates having different chemical composition. Carbohydr. Res..

[64] Morris E.R. (2019). Ordered conformation of xanthan in solutions and “weak gels”: Single helix, double helix—Or both?. Food Hydrocoll..

[65] Matsuda Y., Biyajima Y., Sato T. (2009). Thermal denaturation, renaturation, and aggregation of a double-helical polysaccharide xanthan in aqueous solutions. Polym. J..

[66] Rochas C., Rinaudo M. (1984). Mechanism of gel formation in κ-carrageenan. Biopolymers.

[67] Ciancia M., Milas M., Rinaudo M. (1997). On the specific role of coions and counterions on the κ-carrageenan conformation. Int. J. Biol. Macromol..

[68] Tavagnacco L., Chiessi E., Severini L., Franco S., Buratti E., Capocefalo A., Brasili F., Conte A.M., Missori M., Angelini R. (2023). Molecular origin of the two-step mechanism of gellan aggregation. Sci. Adv..

[69] Brun P., Zamuner A., Battocchio C., Cassari L., Todesco M., Graziani V., Iucci G., Marsotto M., Tortora L., Secchi V. (2021). Bio-functionalized chitosan for bone tissue engineering. Int. J. Mol. Sci..

[70] Ikram R., Mohamed Jan B., Abdul Qadir M., Sidek A., Stylianakis M.M., Kenanakis G. (2021). Recent advances in chitin and chitosan/graphene-based bio-nanocomposites for energetic applications. Polymers.

[71] Yu Y., Xu S., Lic S., Pan H. (2021). Genipin-cross-linked hydrogels based on biomaterials for drug delivery: A review. Biomat. Sci..

[72] Ryu J., Hong S., Lee H. (2015). Bio-inspired adhesive catechol-conjugated chitosan for biomedical applications: A mini review. Acta Biomat..

[73] Do N.H.N., Truong Q.T., Le P.K., Ha A.C. (2022). Recent developments in chitosan hydrogels carrying natural bioactive compounds. Carbohydr. Polym..

[74] Wang M., Lin S., Liu M., Jiao J., Mi H., Sun J., Liu Y., Guo R., Liu S., Fu H. (2023). An injectable and rapidly degraded carboxymethyl chitosan/polyethylene glycol hydrogel for postoperative antiadhesion. Chem. Eng. J..

[75] Liu Z., Xu Y., Su H., Jing X., Wang D., Li S., Chen Y., Guan H., Meng L. (2023). Chitosan-based hemostatic sponges as new generation hemostatic materials for uncontrolled bleeding emergency: Modification, composition, and applications. Carbohydr. Polym..

[76] Fan P., Zeng Y., Zaldivar-Silva D., Agüero L., Wang S. (2023). Chitosan-based hemostatic hydrogels: The concept, mechanism, application, and prospects. Molecules.

[77] Upadhyayaa L., Singhb J., Agarwalc V., Tewaria R.P. (2013). Biomedical applications of carboxymethyl chitosans. Carbohydr. Polym..

[78] Saravanan S., Vimalraj S., Thanikaivelan P., Banudevi S., Manivasagam G. (2019). A review on injectable chitosan/beta glycerophosphate hydrogels for bone tissue regeneration. Int. J. Biol. Macromol..

[79] Suneetha M., Won S.Y., Zo S.M., Han S.S. (2023). Fungal carboxymethyl chitosan-impregnated bacterial cellulose hydrogel as wound-dressing agent. Gels.

[80] Shariatinia Z. (2018). Carboxymethyl chitosan: Properties and biomedical applications. Int. J. Biol. Macromol..

[81] Rao K.M., Narayanan K.B., Uthappa U.T., Park P.H., Choi I., Han S.S. (2022). Tissue adhesive, self-healing, biocompatible, hemostasis, and antibacterial properties of fungal-derived carboxymethyl chitosan-polydopamine hydrogels. Pharmaceutics.

[82] Kwiecie I., Kwiecie M. (2018). Application of polysaccharide-based hydrogels as probiotic delivery systems. Gels.

[83] Lupu A., Rosca I., Gradinaru V.R., Bercea M. (2023). Temperature induced gelation and antimicrobial properties of Pluronic F127 based systems. Polymers.

[84] Hamedi H., Moradi S., Hudson S.M., Tonelli A.E. (2018). Chitosan based hydrogels and their applications for drug delivery in wound dressings: A review. Carbohydr. Polym..

[85] Iftime M.M., Rosca I., Sandu A.S., Marin L. (2022). Chitosan crosslinking with a vanillin isomer toward self-healing hydrogels with antifungal activity. Int. J. Biol. Macromol..

[86] Marinho A., Nunes C., Reis S. (2021). Hyaluronic acid: A key ingredient in the therapy of inflammation. Biomolecules.

[87] Neuman M., Nanau R.M., Oruña-Sanchez L., Coto G. (2015). Hyaluronic acid and wound healing. J. Pharm. Sci..

[88] Burdick J.A., Prestwich G.D. (2011). Hyaluronic acid hydrogels for biomedical applications. Adv. Mater..

[89] Cho W.J., Ahn J., Lee M., Choi H., Park S., Cha K.Y., Lee S.J., Arai Y., Lee S.H. (2023). Combinatorial effect of mesenchymal stem cells and extracellular vesicles in a hydrogel on cartilage regeneration. Tissue Eng. Regen. Med..

[90] Tavianatou A.G., Caon I., Franchi M., Piperigkou Z., Galesso D., Karamanos N.K. (2019). Hyaluronan: Molecular size-dependent signaling and biological functions in inflammation and cancer. FEBS J..

[91] Graca M.F.P., Miguela S.P., Cabrala C.S.D., Correia I.J. (2020). Hyaluronic acid—Based wound dressings: A review. Carbohydr. Polym..

[92] Browne S., Hossainy S., Healy K. (2020). Hyaluronic acid macromer molecular weight dictates the biophysical properties and in vitro cellular response to semisynthetic hydrogels. ACS Biomater. Sci. Eng..

[93] Luo Y., Tan J., Zhou Y., Guo Y., Liao X., He L., Li D., Li X., Li Y. (2023). From crosslinking strategies to biomedical applications of hyaluronic acid-based hydrogels: A review. Int. J. Biol. Macromol..

[94] Ghaffari-Bohlouli P., Simińska-Stanny J., Jafari H., Mirzaei M., Nie L., Delporte C., Shavandi A. (2023). Printable hyaluronic acid hydrogel functionalized with yeast-derived peptide for skin wound healing. Int. J. Biol. Macromol..

[95] Vanoli V., Delleani S., Casalegno M., Pizzetti F., Makvandi P., Haugen H., Mele A., Rossi F., Castiglione F. (2023). Hyaluronic acid-based hydrogels: Drug diffusion investigated by HR-MAS NMR and release kinetics. Carbohydr. Polym..

[96] Galvez-Martin P., Soto-Fernandez C., Romero-Rueda J., Cabañas J., Torrent A., Castells G., Martinez-Puig D. (2023). A novel hyaluronic acid matrix ingredient with regenerative, anti-aging and antioxidant capacity. Int. J. Mol. Sci..

[97] Della Sala F., Longobardo G., Lista G., Messina F., Borzacchiello A. (2023). Effect of hyaluronic acid and mesenchymal stem cells secretome combination in promoting alveolar regeneration. Int. J. Mol. Sci..

[98] Bukhari S.N.A., Roswandi N.L., Waqas M., Habib H., Hussain F., Khan S., Sohail M., Ramli N.A., Thu H.E., Hussain Z. (2018). Hyaluronic acid, a promising skin rejuvenating biomedicine: A review of recent updates and pre-clinical and clinical investigations on cosmetic and nutricosmetic effects. Int. J. Biol. Macromol..

[99] Ahmadian E., Dizaj S.M., Eftekhari A., Dalir E., Vahedi P., Hasanzadeh A., Samiei S. (2020). The potential applications of hyaluronic acid hydrogels in biomedicine. Drug Res..

[100] Pereira H., Sousa D.A., Cunha A., Andrade R., Espregueira-Mendes J., Oliveira M., Reis L.R. (2018). Hyaluronic acid. Adv. Exp. Med. Biol..

[101] Salwowska N., Bebenek K.A., Zadło D., Wcisło-Dziadecka D. (2016). Physiochemical properties and application of hyaluronic acid: A systematic review. J. Cosmet. Dermatol..

[102] Yi X., Xu Z., Liu O., Zhou H., Yuan L., Li D., Zhao L., Mu C., Ge L. (2022). Matrix metalloproteinase-responsive collagen-oxidized hyaluronic acid injectable hydrogels for osteoarthritic therapy. Biomater. Adv..

[103] Lou J., Stowers R., Nam S., Xia Y., Chaudhuri O. (2018). Stress relaxing hyaluronic acid-collagen hydrogels promote cell spreading, fiber remodeling, and focal adhesion formation in 3D cell culture. Biomaterials.

[104] Zhang M., Chen X., Yang K., Dong Q., Yang H., Gu S., Xu W., Zhou Y. (2023). Dual-crosslinked hyaluronic acid hydrogel with self-healing capacity and enhanced mechanical properties. Carbohydr. Polym..

[105] Chun C., Lee D.Y., Kim J.T., Kwon M.K., Kim Y.Z., Kim S.S. (2016). Effect of molecular weight of hyaluronic acid (HA) on viscoelasticity and particle texturing feel of HA dermal biphasic fillers. Biomater. Res..

[106] Weng H., Jia W., Li M., Chen Z. (2022). New injectable chitosan-hyaluronic acid based hydrogels for hemostasis and wound healing. Carbohydr. Polym..

[107] Bai Q., Gao Q., Hu F., Zheng C., Chen W., Sun N., Liu J., Zhang Y., Wu X., Lu T. (2023). Chitosan and hyaluronic-based hydrogels could promote the infected wound healing. Int. J. Biol. Macromol..

[108] Thomas P., Duolikun T., Rumjit N.P., Moosavi S., Lai C.W., Johan M.R.B., Fen L.B. (2020). Comprehensive review on nanocellulose: Recent developments, challenges and future prospects. J. Mech. Behav. Biomed. Mater..

[109] Wohlert M., Benselfelt T., Wågberg L., Furó I., Berglund L.A., Wohlert J. (2022). Cellulose and the role of hydrogen bonds: Not in charge of everything. Cellulose.

[110] Budtova T., Navard P. (2016). Cellulose in NaOH–water based solvents: A review. Cellulose.

[111] Zainal S.H., Mohd N.H., Suhaili N., Anuar F.H., Lazim A.M., Othaman R. (2021). Preparation of cellulose-based hydrogel: A review. J. Mater. Res. Technol..

[112] Chang C., Zhang L., Zhou J., Zhang L., Kennedy J.F. (2010). Structure and properties of hydrogels prepared from cellulose in NaOH/urea aqueous solutions. Carbohydr. Polym..

[113] Barbará P.V., Rafat A.A., Hallet J.P., Brandt-Talbot A. (2023). Purifying cellulose from major waste streams using ionic liquids and deep eutectic solvents. Curr. Opin. Green Sustain. Chem..

[114] Ciolacu D.E., Nicu R., Ciolacu F. (2020). Cellulose-based hydrogels as sustained drug-delivery systems. Materials.

[115] Kim S., Song M., Lee M., Kwon S. (2023). Controlled release of quercetin from HPMC/gellan gum hydrogel for inhibiting melanogenesis in murine melanoma cells. Korean J. Chem. Eng..

[116] El Fawal G.F., Abu-Serie M.M., Hassan M.A., Elnouby M.S. (2018). Hydroxyethyl cellulose hydrogel for wound dressing: Fabrication, characterization and in vitro evaluation. Int. J. Biol. Macromol..

[117] Li K.T., Nguyen C.T., Lac T.D., Nguyen L.G.T., Tran T.L., Tran-Van H. (2023). Facilely preparing carboxymethyl chitosan/hydroxyethyl cellulose hydrogel films for protective and sustained release of fibroblast growth factor 2 to accelerate dermal tissue repair. J. Drug Deliv. Sci. Technol..

[118] Huang S., Su S., Gan H., Wu L., Lin C., Xu D., Zhou H., Lin X., Qion Y. (2019). Facile fabrication and characterization of highly stretchable lignin-based hydroxyethyl cellulose self-healing hydrogel. Carbohydr. Polym..

[119] Alam M.N., Islam M.S., Christopher L.P. (2019). Sustainable production of cellulose-based hydrogels with superb absorbing potential in physiological saline. ACS Omega.

[120] Kaur P., Bohidar H.B., Nisbet D.R., Pfeffer F.M., Rifai A., ·Williams R., Agrawal R. (2023). Waste to high-value products: The performance and potential of carboxymethylcellulose hydrogels via the circular economy. Cellulose.

[121] Hameed A., Khurshidm S., Adnanm A. (2020). Synthesis and characterization of carboxymethyl cellulose based hydrogel and its applications on water treatment. Desalin. Water Treat..

[122] Gabriel G.R., Gococo B.G., Yu C.A., Nalzaro P.J., Tumolva T. (2020). Synthesis and characterization of sodium carboxymethyl cellulose/sodium alginate/hydroxypropyl cellulose hydrogel for agricultural water storage and controlled nutrient release. Solid State Phenom..

[123] Kanikireddy V., Varaprasad K., Jayaramudu T., Karthikeyan C., Sadiku R. (2020). Carboxymethyl cellulose-based materials for infection control and wound healing: A review. Int. J. Biol. Macromol..

[124] Dacrory S., Abou-Yousef H., Abou-Zeid R.E., Kamel S., Abdel-Aziz M.S., Elbadry M. (2018). Preparation and characterization of eco-friendly carboxymethyl cellulose antimicrobial nanocomposite hydrogels. J. Renew. Mater..

[125] Bercea M., Navard P. (2016). Comparison of elasticity contributions during the flow of a cellulose derivative solution. Cell. Chem. Technol..

[126] Ogawa A., Nakayama S., Uehara M., Mori Y., Takahashi M., Aiba T., Kurosaki Y. (2014). Pharmaceutical properties of a low-substituted hydroxypropyl cellulose (L-HPC) hydrogel as a novel external dressing. Int. J. Pharm..

[127] Yi X., Cheng F., Wei X., Li H., Qian J., He J. (2023). Bioinspired adhesive and self-healing bacterial cellulose hydrogels formed by a multiple dynamic crosslinking strategy for sealing hemostasis. Cellulose.

[128] Zhang K., Wu D., Chang L., Duan W., Wang Y., Li W., Qion J. (2023). Cellulose based self-healing hydrogel through boronic ester connections for wound healing and antitumor applications. Int. J. Biol. Macromol..

[129] Cuenca E., Postolachi V., Ferrara L. (2023). Cellulose nanofibers to improve the mechanical and durability performance of self-healing ultra-highperformance concretes exposed to aggressive waters. Constr. Build. Mater..

[130] Tang J., Javaid M.U., Pan C., Yu G., Berry R.M., Tam R.C. (2020). Self-healing stimuli-responsive cellulose nanocrystal hydrogels. Carbohydr. Polym..

[131] Baron R.I., Bercea M., Avadanei M., Lisa G., Biliuta G., Coseri S. (2019). Green route for the fabrication of self-healable hydrogels based on tricarboxy cellulose and poly(vinyl alcohol). Int. J. Biol. Macromol..

[132] Darie R.N., Bercea M., Kozlowski M., Spiridon I. (2011). Evaluation of properties of LDPE/OAK wood composites exposed to artificial ageing. Cell. Chem. Technol..

[133] Bercea M., Navard P. (2000). Shear dynamics of aqueous suspensions of cellulose whiskers. Macromolecules.

[134] Nyamayro K., Mehrkhlodavandi P., Hatzikiriakos S.G. (2023). Impact of counterion valency on the rheology of sulfonated cellulose nanocrystal hydrogels. Carbohydr. Polym..

[135] Ojagh S.M.A., Amini M., Cranmer-Smith C., Vahabzadeh F., Arjmand M., Tam K.C., Rojas O.J., Kamkar M., van de Ven T.G.M. (2023). Crystalline and hairy nanocelluloses for 3D printed hydrogels and strongly structured cryogels. ACS Sustain. Chem. Eng..

[136] Zhang Z., Abidi N., Lucia L., Chabi S., Denny C.T., Parajuli P., Rumi S.S. (2023). Cellulose/nanocellulose superabsorbent hydrogels as a sustainable platform for materials applications: A mini-review and perspective. Carbohydr. Polym..

[137] Solhi L., Guccini V., Heise K., Solala I., Niinivaara E., Xu W., Mihhels K., Kröger M., Meng Z., Wohlert J. (2023). Understanding nanocellulose–water interactions turning a detriment into an asset. Chem. Rev..

[138] He X., Lu Q. (2023). Design and fabrication strategies of cellulose nanocrystal-based hydrogel and its highlighted application using 3D printing: A review. Carbohydr. Polym..

[139] Pan X., Li J., Ma N., Ma X., Gao M. (2023). Bacterial cellulose hydrogel for sensors. Chem. Eng. J..

[140] Bercea M. (2023). Rheology as a tool for fine-tuning the properties of printable bioinspired gels. Molecules.

[141] Norizan M.N., Shazleen S.S., Alias A.H., Sabaruddin F.A., Asyraf M.R.M., Zainudin E.S., Abdullah N., Samsudin M.S., Kamarudin S.H., Norrrahim M.N.F. (2022). Nanocellulose-based nanocomposites for sustainable applications: A review. Nanomaterials.

[142] Gomes D., Batista-Silva J.P., Sousa A., Passarinha L.A. (2023). Progress and opportunities in gellan gum-based materials: A review of preparation, characterization and emerging applications. Carbohydr. Polym..

[143] Gonçalves V.M.F., Reis A., Domingues M.R.M., Lopes-da-Silva J.A., Fialho A.M., Moreira L.M., Sá-Correia I., Coimbra M.A. (2009). Structural analysis of gellans produced by Sphingomonas elodea strains by electrospray tandem mass spectrometry. Carbohydr. Polym..

[144] Morris E.R., Nishinari K., Rinaudo M. (2012). Gelation of gellan—A review. Food Hydrocoll..

[145] Annaka M. (2023). Anion specific conformational change in aqueous gellan gum solutions. Carbohydr. Polym..

[146] Chen Q., Ma H., Yuan Y., Han X., Zhu J., Zhang H. (2017). Rheological behavior of high acyl gellan gum solution at gel point. Int. J. Food Prop..

[147] García M.C., Trujillo L.A., Muñoz J., Alfaro M.C. (2018). Gellan gum fluid gels influence of the nature and concentration of gel-promoting ions on rheological properties. Colloid Polym. Sci..

[148] Grasdalen H., Smidsrød O. (1987). Gelation of gellan gum. Carbohydr. Polym..

[149] Lorenzo G., Zaritzky N., Califano A. (2013). Rheological analysis of emulsion-filled gels based on high acyl gellan gum. Food Hydrocoll..

[150] Chen T., Wu Y., Loi F., Zhang N., Yan B., Zhao J., Zhang H., Chen W., Fan D. (2022). Unusual gelation behavior of low-acetyl gellan under microwave field: Changes in rheological and hydration properties. Carbohydr. Polym..

[151] Das M., Giri T.K. (2020). Hydrogels based on gellan gum in cell delivery and drug delivery. J. Drug Deliv. Sci. Technol..

[152] Mendes A.I., Fraga A.G., Peixoto M.J., Aroso I., Longatto-Filho A., Marques A.P., Pedrosa J. (2023). Gellan gum spongy-like hydrogel-based dual antibiotic therapy for infected diabetic wounds. Bioeng. Transl. Med..

[153] Muthukumar T., Song J.E., Khang G. (2019). Biological role of gellan gum in improving scaffold drug delivery, cell adhesion properties for tissue engineering applications. Molecules.

[154] Cernencu A.I., Ionita M. (2023). The current state of the art in gellan-based printing inks in tissue engineering. Carbohydr. Polym..

[155] Baek J.S., Carlomagno C., Muthukumar T., Kim D., Park J.H., Song J.E., Migliaresi C., Motta A., Reis R.L., Khang G. (2019). Evaluation of cartilage regeneration in gellan gum/agar blended hydrogel with improved injectability. Macromol. Res..

[156] Bacelar A.H., Silva-Correia J., Oliveira J.M., Reis R.L. (2016). Recent progress in gellan gum hydrogels provided by functionalization strategies. J. Mater. Chem. B.

[157] Xu Z., Li Z., Jiang S., Bratlie K.M. (2018). Chemically modified gellan gum hydrogels with tunable properties for use as tissue engineering scaffolds. ACS Omega.

[158] Mouser V.H.M., Melchels F.P.W., Visser J., Dhert W.J.A., Gawlitta D., Malda J. (2016). Yield stress determines bioprintability of hydrogels based on gelatin-methacryloyl and gellan gum for cartilage bioprinting. Biofabrication.

[159] Compaan A.M., Song K., Huang Y. (2019). Gellan fluid gel as a versatile support bath material for fluid extrusion bioprinting. ACS Appl. Mater. Interfaces.

[160] Richa, Choudhury A.R. (2023). Self-assembled pH-stable gellan/κ-carrageenan bigel: Rheological studies and viscosity prediction by neural network. Int. J. Biol. Macromol..

[161] Naji-Tabasi S., Shahidi-Noghabi M., Dovom A.M. (2023). Investigating the fabrication and functional properties of new composite hydrogels containing gellan/alginate/xanthan gum. J. Sol-Gel Sci. Technol..

[162] Pan Z., Song C., Chen Y., Qian X. (2019). Locust bean gum/gellan gum double-network hydrogels with superior self-healing and pH-driven shape-memory properties. Soft Matter.

[163] Hernández L.R., Hernández N.C., Tecante A., López-Ortega M.A., Cuellar M.R.L., Rodrígues-Hernández A.I. (2023). Mixed gels based on low acyl gellan and citrus pectin: A linear viscoelastic analysis. Food Hydrocoll..

[164] Tran Vo T.M., Kobayashi T., Potiyaraj P. (2022). Viscoelastic analysis of pectin hydrogels regenerated from citrus pomelo waste by gelling effects of calcium ion crosslinking at different pHs. Gels.

[165] Fan Z., Cheng P., Gao Y., Wang D., Jia G., Zhang P., Han J. (2022). Understanding the rheological properties of a novel composite salecan/gellan hydrogels. Food Hydrocoll..

[166] Nita L.E., Croitoriu A., Serban A.M., Bercea M., Rusu A.G., Ghilan A., Butnaru M., Mititelu-Tartau L., Chiriac A.P. (2023). New hydrogels based on agarose/phytagel and peptides. Macromol. Biosci..

[167] Liu L., Zhang D., Song X., Guo M., Wang Z., Geng F., Zhou X., Nie S. (2022). Compound hydrogels derived from gelatin and gellan gum regulates the release of anthocyanins in simulated digestion. Food Hydrocoll..

[168] Habibi H., Khosravi-Darani K. (2017). Effective variables on production and structure of xanthan gum and its food applications: A review. Biocatal. Agric. Biotechnol..

[169] Zhao L., Pan F., Mehmood A., Zhang Y.L., Hao S., Rehman A.U., Li J.Y., Wang C.T., Wang Y. (2020). Protective effect and mechanism of action of xanthan gum on the color stability of black rice anthocyanins in model beverage systems. Int. J. Biol. Macromol..

[170] Jana A.K., Ghosh P. (1999). Effect of citric acid on the biosynthesis and composition of xanthan. J. Gen. Appl. Microbiol..

[171] Davidson I.W. (1978). Production of polysaccharide by Xanthomonas campestris in continuous culture. FEMS Microbiol. Lett..

[172] Kool M.M., Gruppen H., Sworn G., Schols H.A. (2013). Comparison of xanthans by the relative abundance of its six constituent repeating units. Carbohydr. Polym..

[173] Kool M.M., Gruppen H., Sworn G., Schols H.A. (2014). The influence of the six constituent xanthan repeating units on the order–disorder transition of xanthan. Carbohydr. Polym..

[174] Callet F., Milas M., Rinaudo M. (1987). Influence of acetyl and pyruvate contents on rheological properties of xanthan in dilute solution. Int. J. Biol. Macromol..

[175] Bercea M., Morariu S. (2020). Real-time monitoring the order-disorder conformational transition of xanthan gum. J. Mol. Liq..

[176] Brunchi C.-E., Avadanei M., Bercea M., Morariu S. (2019). Chain conformation of xanthan in solution as influenced by temperature and salt addition. J. Mol. Liq..

[177] Tako M., Nakamura S. (1989). Evidence for intramolecular associations in xanthan molecules. Agric. Biol. Chem..

[178] Rochefort W.E., Middleman S. (1987). Rheology of xanthan gum: Salt, temperature, and strain effects in oscillatory and steady shear experiments. J. Rheol..

[179] Muller G., Anrhourrache M., Lecourtier J., Chauveteau G. (1986). Salt dependence of the conformation of a single-stranded xanthan. Int. J. Biol. Macromol..

[180] Abbaszadeh A.H., Lad M., Morris G.A., MacNaughtan W., Sworn G., Foster T.J. (2019). The influence of charge on the multiple thermal transitions observed in xanthan. Food Hydrocoll..

[181] N’gouamba E., Essadik M., Goyon J., Oerther T., Coussot P. (2021). Yielding and rheopexy of aqueous xanthan gum solutions. Rheol. Acta.

[182] Jadav M., Pooja D., Adams D.J., Kulhari H. (2023). Advances in xanthan gum-based systems for the delivery of therapeutic agents. Pharmaceutics.

[183] Fantou C., Roy A.N., Dé E., Comesse S., Grisel M.I., Renou F. (2017). Chemical modification of xanthan in the ordered and disordered states: An open route for tuning the physico-chemical properties. Carbohydr. Polym..

[184] Fujiwara J., Iwanami T., Takahashi M., Tanaka R., Hatakeyama T., Hatakeyama H. (2000). Structural change of xanthan gum association in aqueous solutions. Thermochim. Acta.

[185] Wang C.S., Natale G., Virgilio N., Heuzey M.C. (2016). Synergistic gelation of gelatin B with xanthan gum. Food Hydrocoll..

[186] Wang C.S., Natale G., Virgilio N., Wood-Adams P., Heuzey M.C. (2017). A mechanism for the synergistic gelation properties of gelatin B and xanthan gum aqueous mixtures. Carbohydr. Polym..

[187] Martínez-Ruvalcaba A., Chornet E., Rodrigue D. (2007). Viscoelastic properties of dispersed chitosan/xanthan hydrogels. Carbohydr. Polym..

[188] Martınez-Padilla L.P., López-Araiza F., Tecante A. (2004). Steady and oscillatory shear behavior of fluid gels formed by binary mixtures of xanthan and gellan. Food Hydrocoll..

[189] Caldera-Villalobos M., Claudio-Rizo J.A., Cabrera-Munguía D.A., Becerra-Rodriguez J.J., Soriano-Corral F., Herrera-Guerrero A. (2023). Smart collagen/xanthan gum-based hydrogels with antibacterial effect, drug release capacity and excellent performance in vitro bioactivity for wound healing application. Biomed. Mater..

[190] Cofelice M., Messia M.C., Marconi W., Cuomo F., Lopez F. (2023). Effect of the xanthan gum on the rheological properties of alginate hydrogels. Food Hydrocoll..

[191] Baniasadi H., Kimiaei E., Teixeira Polez R., Ajdary R., Rojas O.J., Österberg M., Seppälä J. (2022). High-resolution 3D printing of xanthan gum/nanocellulose bio-inks. Int. J. Biol. Macromol..

[192] Abka-khajouei R., Tounsi L., Shahabi N., Patel A.K., Abdelkafi S., Michaud P. (2022). Structures, properties and applications of alginates. Mar. Drugs.

[193] Lee H.P., Gu L., Mooney D.J., Levenston M.E., Chaudhuri O. (2017). Mechanical confinement regulates cartilage matrix formation by chondrocytes. Nat. Mater..

[194] Hu C., Lu W., Mata A., Nishinari K., Fang Y. (2021). Ions-induced gelation of alginate: Mechanisms and applications. Int. J. Biol. Macromol..

[195] Johnson K.-A., Muzzin N., Toufanian S., Slick R.A., Lawlor M.W., Seifried B., Moquin P., Latulippe D., Hoare T. (2020). Drug-impregnated, pressurized gas expanded liquid-processed alginate hydrogel scaffolds for accelerated burn wound healing. Acta Biomater..

[196] Stokke B.T., Smidsrød O., Bruheim P., Skjåk-Bræk G. (1991). Distribution of uronate residues in alginate chains in relation to alginate gelling properties. Macromolecules.

[197] Stokke B.T., Smidsrød O., Bruheim P., Zanetti F., Strand W., Skjåk-Bræk G. (1993). Distribution of uronate residues in alginate chains in relation to alginate gelling properties—2: Enrichment of β-D-mannuronic acid and depletion of α-L-guluronic acid in sol fraction. Carbohydr. Polym..

[198] Charbonier F., Indana D., Chaudhuri O. (2021). Tuning viscoelasticity in alginate hydrogels for 3D cell culture studies. Curr. Protoc..

[199] Yang J.-S., Xie Y.-J., He W. (2011). Research progress on chemical modification of alginate: A review. Carbohydr. Polym..

[200] Martinsen A., Skjak-Braek G., Smidsrød O. (1987). Alginate as immobilization material: I. Correlation between chemical and physical properties of alginate gel beads. Biotechnol. Bioeng..

[201] Draget K.I., Braek G.S., Smidsrød O. (1994). Alginic acid gels—The effect of alginate chemical-composition and molecular-weight. Carbohydr. Polym..

[202] Stokke B.T., Draget K.I., Smidsrød O., Yuguchi Y., Urakawa H., Kajiwara K. (2000). Small-angle X-ray scattering and rheological characterization of alginate gels. 1. Ca-alginate gels. Macromolecules.

[203] Ramos P.E., Silva P., Alario M.M., Pastrana L.M., Teixeira J.A., Cerqueira M.A., Vicente A.A. (2018). Effect of alginate molecular weight and M/G ratio in beads properties foreseeing the protection of probiotics. Food Hydrocoll..

[204] Hecht H., Srebnik S. (2016). Structural characterization of sodium alginate and calcium alginate. Biomacromolecules.

[205] Bennacef C., Desobry-Banon S., Probst L., Desobry S. (2021). Advances on alginate use for spherification to encapsulate biomolecules. Food Hydrocoll..

[206] Menakbi C., Quignard F., Mineva T. (2016). Complexation of trivalent metal cations to mannuronate type alginate models from a density functional study. J. Phys. Chem. B.

[207] Mørch Ý.A., Donati I., Strand B.L., Skjåk-Bræk G. (2006). Effect of Ca^2+^, Ba^2+^, and Sr^2+^ on alginate microbeads. Biomacromolecules.

[208] Grant G.T., Morris E.R., Rees D.A., Smith P.J.C., Thom D. (1973). Biological interactions between polysaccharides and divalent cations: The egg-box model. FEBS Lett..

[209] Braccini I., Perez S. (2001). Molecular basis of Ca^2+^-induced gelation in alginates and pectins: The egg-box model revisited. Biomacromolecules.

[210] Cao L., Lu W., Mata A., Nishinari K., Fang Y. (2020). Egg-box model-based gelation of alginate and pectin: A review. Carbohydr. Polym..

[211] Feng L., Cao Y., Xu D., Wang S., Zhang J. (2017). Molecular weight distribution, rheological property and structural changes of sodium alginate induced by ultrasound. Ultrason. Sonochem..

[212] Fu S., Thacker A., Sperger D.M., Boni R.L., Buckner I.S., Velankar S., Munson E.J., Block L.H. (2011). Relevance of rheological properties of sodium alginate in solution to calcium alginate gel properties. AAPS PharmSciTech.

[213] Wang Y., Zhao Y., He J., Sun C., Lu W., Zhang Y., Fang Y. (2023). Doubling growth of egg-box structure during Calcium-mediated molecular assembly of alginate. J. Colloid Interface Sci..

[214] Donati I., Holtan S., Mørch Y.A., Borgogna M., Dentini M., Skjåk-Bræk G. (2005). New hypothesis on the role of alternating sequences in calcium—Alginate gels. Biomacromolecules.

[215] Fang Y., Al-Assaf S., Phillips G.O., Nishinari K., Funami T., Williams P.A., Li L. (2007). Multiple steps and critical behaviors of the binding of calcium to alginate. J. Phys. Chem. B.

[216] Borgogna M., Skjåk-Bræk G., Paoletti S., Donati I. (2013). On the initial binding of alginate by calcium ions. The tilted egg-box hypothesis. J. Phys. Chem. B.

[217] Sikorski P., Mo F., Skjåk-Bræk G., Stokke B.T. (2007). Evidence for egg-box-compatible interactions in calcium-alginate gels from fiber X-ray diffraction. Biomacromolecules.

[218] Wang H., Wan Y., Wang W., Li W., Zhu J. (2018). Effect of calcium ions on the III steps of self-assembly of SA investigated with atomic force microscopy. Int. J. Food Prop..

[219] Lerbret A., Assifaoui A. (2022). How accurate is the egg-box model in describing the binding of calcium to polygalacturonate? A molecular dynamics simulation study. J. Phys. Chem. B.

[220] Besiri I.N., Goudoulas T.B., Germann N. (2020). Custom-made rheological setup for in situ real-time fast alginate-Ca^2+^ gelation. Carbohydr. Polym..

[221] Liu X., Qian L., Shu T., Tong Z. (2003). Rheology characterization of sol-gel transition in aqueous alginate solutions induced by calcium cations through in situ release. Polymer.

[222] Chan L.W., Ying L.H., Heng P.W.S. (2006). Mechanisms of external and internal gelation and their impact on the functions of alginate as a coat and delivery system. Carbohydr. Polym..

[223] Sadeghi D., Solouk A., Samadikuchaksaraei A., Seifalian A.M. (2021). Preparation of internally-crosslinked alginate microspheres: Optimization of process parameters and study of pH-responsive behaviors. Carbohydr. Polym..

[224] Lopez-Sanchez P., Assifaoui A., Cousin F., Moser J., Bonilla M.R., Ström A. (2022). Impact of glucose on the nanostructure and mechanical properties of calcium-alginate hydrogels. Gels.

[225] Mahdi M.H., Diryak R., Kontogiorgos V., Morris G.A., Smith A.M. (2016). In situ rheological measurements of the external gelation of alginate. Food Hydrocoll..

[226] Drury J.L., Dennis R.G., Mooney D.J. (2004). The tensile properties of alginate hydrogels. Biomaterials.

[227] Wang M., Chen L., Zhang Z. (2021). Potential applications of alginate oligosaccharides for biomedicine—A mini review. Carbohydr. Polym..

[228] Mierke C.T. (2021). Viscoelasticity acts as a marker for tumor extracellular matrix characteristics. Front. Cell Dev. Biol..

[229] Indana D., Agarwal P., Bhutani N., Chaudhuri O. (2021). Viscoelasticity and adhesion signaling in biomaterials control human pluripotent stem cell morphogenesis in 3D culture. Adv. Mater..

[230] Chaudhuri O., Cooper-White J., Janmey P.A., Mooney D.J., Shenoy V.B. (2020). The impact of extracellular matrix viscoelasticity on cellular behavior. Nature.

[231] Hwang S.D., Sim S.B., Cha H.J. (2007). Cell adhesion biomaterial based on mussel adhesive protein fused with RGD peptide. Biomaterials.

[232] Feng Y., Mrksich M. (2004). The synergy peptide PHSRN and the adhesion peptide RGD mediate cell adhesion through a common mechanism. Biochemistry.

[233] Yang Y.J., Kwon Y., Choi B.H., Jung D., Seo J.H., Lee K.H., Cha H.J. (2014). Multifunctional adhesive silk fibroin with blending of RGD-bioconjugated mussel adhesive protein. Biomacromolecules.

[234] Jeon O., Alsberg E. (2013). Photofunctionalization of alginate hydrogels to promote adhesion and proliferation of human mesenchymal stem cells. Tissue Eng. Part A.

[235] Abasalizadeh F., Moghaddam S.V., Alizadeh E., Akbari E., Kashani E., Fazljou S.M.B., Torbati M., Akbarzadeh A. (2020). Alginate-based hydrogels as drug delivery vehicles in cancer treatment and their applications in wound dressing and 3D bioprinting. J. Biol. Eng..

[236] Puscaselu R.G., Lobiuc A., Dimian M., Covasa M. (2020). Alginate: From food industry to biomedical applications and management of metabolic disorders. Polymers.

[237] Abbasi A.R., Sohail M., Minhas M.U., Khaliq T., Kousar M., Khan S., Hussain Z., Munir A. (2020). Bioinspired sodium alginate based thermosensitive hydrogel membranes for accelerated wound healing. Int. J. Biol. Macromol..

[238] Miguel S.P., Cabral C.S.D., Moreira A.F., Correia I.J. (2019). Production and characterization of a novel asymmetric 3D printed construct aimed for skin tissue regeneration. Colloids Surf. B.

[239] Choi Y., Park K., Choi H., Son D., Shin M. (2021). Self-healing, stretchable, biocompatible, and conductive alginate hydrogels through dynamic covalent bonds for implantable electronics. Polymers.

[240] Sharma A., Chetna Verma C., Mukhopadhyay S., Gupta A., Gupta B. (2022). Development of sodium alginate/glycerol/tannic acid coated cotton as antimicrobial system. Int. J. Biol. Macromol..

[241] Kostenko A., Swioklo S., Connon C.J. (2022). Effect of calcium sulphate precrosslinking on rheological parameters of alginate based bio-inks and on human corneal stromal fibroblast survival in 3D bio-printed constructs. Front. Mech. Eng..

[242] Leone G., Torricelli P., Chiumiento A., Facchini A., Barbucci R. (2008). Amidic alginate hydrogel for nucleus pulposus replacement. J. Biomed. Mater. Res..

[243] Jarrah R., Sammak S.E., Onyedimma C., Ghaith A.K., Moinuddin F.M., Bhandarkar A.R., Siddiqui A., Madigan N., Bydon M. (2022). The role of alginate hydrogels as a potential treatment modality for spinal cord injury: A comprehensive review of the literature. Neurospine.

[244] Lee K.Y., Mooney D.J. (2012). Alginate: Properties and biomedical applications. Prog. Polym. Sci..

[245] Geng Z., Ji Y., Yu S., Liu Q., Zhou Z., Guo C., Lu D., Pei D. (2021). Preparation and characterization of a dual cross-linking injectable hydrogel based on sodium alginate and chitosan quaternary ammonium salt. Carbohydr. Res..

[246] Cui L., Hu J., Wang W., Yan C., Guo Y., Tu C. (2020). Smart pH response flexible sensor based on calcium alginate fibers incorporated with natural dye for wound healing monitoring. Cellulose.

[247] Han C., Wang X., Ni Z., Ni Y., Huan W., Lv Y., Bai S. (2020). Effects of nanocellulose on alginate/gelatin bio-inks for extrusion-based 3D printing. BioResources.

[248] Chen H., Xing X., Tan H., Jia Y., Zhou T., Chen Y., Ling Z., Hu X. (2017). Covalently antibacterial alginate-chitosan hydrogel dressing integrated gelatin microspheres containing tetracycline hydrochloride for wound healing. Mater. Sci. Eng. C.

[249] Serafin A., Culebras M., Collins M.N. (2023). Synthesis and evaluation of alginate, gelatin, and hyaluronic acid hybrid hydrogels for tissue engineering applications. Int. J. Biol. Macromol..

[250] Im S., Choe G., Seok J.M., Yeo S.J., Lee J.H., Kim W.D., Lee J.Y., Park S.A. (2022). An osteogenic bioink composed of alginate, cellulose nanofibrils, and polydopamine nanoparticles for 3D bioprinting and bone tissue engineering. Int. J. Biol. Macromol..

[251] Campo V.L., Kawano D.F., Silva D.B., Carvalho D.I. (2009). Carrageenans: Biological properties, chemical modifications and structural analysis—A review. Carbohydr. Polym..

[252] Li L., Ni R., Shao Y., Mao S. (2014). Carrageenan and its applications in drug delivery. Carbohydr. Polym..

[253] Wang Y., Yuan C., Cui B., Liu Y. (2018). Influence of cations on texture, compressive elastic modulus, sol-gel transition and freeze-thaw properties of kappa-carrageenan gel. Carbohydr. Polym..

[254] Kara S., Tamerler C., Bermek H., Pekcan N. (2003). Cation effects on sol–gel and gel–sol phase transitions of κ-carrageenan–water system. Int. J. Biol. Macromol..

[255] Borgström J., Piculell L., Viebke C., Talmon Y. (1996). On the structure of aggregated kappa-carrageenan helices. A study by cryo-TEM, optical rotation and viscometry. Int. J. Biol. Macromol..

[256] Piculell L., Borgström J., Chronakis I.S., Quist P.-O., Viebke C. (1997). Organisation and association of κ-carrageenan helices under different salt conditions. Int. J. Biol. Macromol..

[257] Chronakis I.S., Piculell L., Borgström J. (1996). Rheology of kappa-carrageenan in mixtures of sodium and cesium iodide: Two types of gels. Carbohydr. Polym..

[258] Robal M., Brenner T., Matsukawa S., Ogawa H., Truus K., Rudolph B., Tuvikene R. (2017). Monocationic salts of carrageenans: Preparation and physico-chemical properties. Food Hydrocoll..

[259] Rochas C., Rinaudo M. (1980). Activity coefficients of counterions and conformation in kappa-carrageenan systems. Biopolymers.

[260] Evageliou V.I., Ryan P.M., Morris E.R. (2019). Effect of monovalent cations on calcium-induced assemblies of kappa carrageenan. Food Hydrocoll..

[261] Morris E.R., Rees D.A., Robinson G. (1980). Cation-specific aggregation of carrageenan helices: Domain model of polymer gel structure. J. Mol. Biol..

[262] Elangwe C.N., Morozkina S.N., Olekhnovich R.O., Polyakova V.O., Krasichkov A., Yablonskiy P.K., Uspenskaya M.V. (2023). Pullulan-based hydrogels in wound healing and skin tissue engineering applications: A review. Int. J. Biol. Macromol..

[263] Bercea M., Plugariu I.A., Gradinaru L.-M., Avadanei M., Doroftei F., Gradinaru V.R. (2023). Hybrid hydrogels for neomycin delivery: Synergistic effects of natural/synthetic polymers and proteins. Polymers.

[264] Bercea M., Constantin M., Plugariu I.A., Daraba M.O., Ichim D.L. (2022). Thermosensitive gels of pullulan and Poloxamer 407 as potential injectable biomaterials. J. Mol. Liq..

[265] Ganie S.A., Rather L.J., Li Q. (2021). A review on anticancer applications of pullulan and pullulan derivative nanoparticles. Carbohydr. Polym. Technol. Appl..

[266] Raychaudhuri R., Naik S., Shreya A.B., Neha Kandpal N., Pandey A., Kalthur G., Mutalik S. (2020). Pullulan based stimuli responsive and sub cellular targeted nanoplatforms for biomedical application: Synthesis, nanoformulations and toxicological perspective. Int. J. Biol. Macromol..

[267] Grigoras A.G. (2019). Drug delivery systems using pullulan, a biocompatible polysaccharide produced by fungal fermentation of starch. Environ. Chem. Lett..

[268] Su T., Wu L., Pan X., Zhang C., Shi M., Gao R., Qi X., Dong W. (2019). Pullulan-derived nanocomposite hydrogels for wastewater remediation: Synthesis and characterization. J. Colloid Interface Sci..

[269] Bercea M., Plugariu I.A. (2020). Associative interactions between pullulan and negatively charged bovine serum albumin in physiological saline solutions. Carbohydr. Polym..

[270] Wang Z., Li K.Y., Xu Q.R., Fu G.L., Li H.Y., Yang W.Z. (2023). Preparation and evaluation of chitosan- and hyaluronic acid-grafted pullulan succinate films for skin wound healing. Int. J. Biol. Macromol..

[271] Le Guilcher C., Merlen G., Dellaquila A., Labour M.N., Aid R., Tordjmann T., Letourneur D., Simon-Yarza T. (2023). Engineered human liver based on pullulan-dextran hydrogel promotes mice survival after liver failure. Mat. Today Bio.

[272] Pandeirada C.O., Achterweust M., Janssen H.G., Westphal Y., Schols H.A. (2022). Periodate oxidation of plant polysaccharides provides polysaccharide-specific oligosaccharides. Carbohydr. Polym..

[273] Nypelö T., Berke B., Spirk S., Sirviö J.A. (2021). Review: Periodate oxidation of wood polysaccharides—Modulation of hierarchies. Carbohydr. Polym..

[274] Pierre G., Punta C., Delattre C., Melone L., Dubessay P., Fiorati A., Pastori N., Galante Y.M., Michaud P. (2017). TEMPO-mediated oxidation of polysaccharides an ongoing story. Carbohydr. Polym..

[275] Coseri S., Bercea M., Harabagiu V., Budtova T. (2016). Oxidation vs. degradation in polysaccharides: Pullulan—A case study. Eur. Polym. J..

[276] Constantin M., Cosman B., Bercea M., Ailiesei G.-L., Fundueanu G. (2022). Thermosensitive poloxamer-graft-carboxymethyl pullulan: A potential injectable hydrogel for drug delivery. Polymers.

[277] Popescu I., Constantin M., Bercea M., Cosman B.-P., Suflet D.M., Fundueanu G. (2023). Poloxamer/carboxymethyl pullulan aqueous systems—Miscibility and thermogelation studies using viscometry, rheology and dynamic light scattering. Polymers.

[278] Su T., Zhao W., Wu L., Dong W., Qi X. (2020). Facile fabrication of functional hydrogels consisting of pullulan and polydopamine fibers for drug delivery. Int. J. Biol. Macromol..

[279] Constantin M., Bucatariu S., Sacarescu L., Daraba O.M., Anghelache M., Fundueanu G. (2020). Pullulan derivative with cationic and hydrophobic moieties as an appropriate macromolecule in the synthesis of nanoparticles for drug delivery. Int. J. Biol. Macromol..

[280] Bercea M., Biliuta G., Avadanei M., Baron R.I., Butnaru M., Coseri S. (2019). Self-healing hydrogels of oxidized pullulan and poly(vinyl alcohol). Carbohydr. Polym..

[281] Zhang L., Liu J., Zheng X., Zhang A., Zhang X., Tang K. (2019). Pullulan dialdehyde crosslinked gelatin hydrogels with high strength for biomedical applications. Carbohydr. Polym..

[282] Rui Q., Gao J., Yin Z.Z., Li J.Y., Cai W.R., Wu D.T., Kong Y. (2023). A biodegradable pH and glutathione dual-triggered drug delivery system based on mesoporous silica, carboxymethyl chitosan and oxidized pullulan. Int. J. Biol. Macromol..

[283] Verma R., Singh V., Koch B., Kumar M. (2023). Evaluation of methotrexate encapsulated polymeric nanocarrier for breast cancer treatment. Colloids Surf. B.

[284] Simpson L.W., Good T.A., Leach J.B. (2020). Protein folding and assembly in confined environments: Implications for protein aggregation in hydrogels and tissues. Biotechnol. Adv..

[285] Nie J., Zhang X., Wang W., Ren J., Zeng A.P. (2021). Tunable protein hydrogels: Present State and Emerging Development. Tunable Hydrogels.

[286] https://www.rcsb.org/structure/5CTI.

[287] https://www.rcsb.org/structure/6ec0.

[288] Lockhart-Cairns M.P., Newandee H., Thomson J., Weiss A.S., Baldock C., Tarakanova A. (2020). Transglutaminase-mediated cross-linking of tropoelastin to fibrillin stabilises the elastin precursor prior to elastic fibre assembly. J. Mol. Biol..

[289] Meganathan I., Sundarapandian A., Shanmugam G., Ayyadurai N. (2022). Three-dimensional tailor-made collagen-like proteins hydrogel for tissue engineering applications. Biomat. Adv..

[290] Sapudom J., Kalbitzer L., Wu X., Martin S., Kroy K., Pompe T. (2019). Fibril bending stiffness of 3D collagen matrices instructs spreading and clustering of invasive and non-invasive breast cancer cells. Biomaterials.

[291] Walsh A.J., Cook R.S., Lee J.H., Arteaga C.L., Skala M.C. (2015). Collagen density and alignment in responsive and resistant trastuzumab-treated breast cancer xenografts. J. Biomed. Opt..

[292] Mouw J.K., Yui Y., Damiano L., Bainer R.O., Lakins J.N., Acerbi I., Ou G., Wijekoon A.C., Levental K.R., Gilbert P.M. (2014). Tissue mechanics modulate microRNA-dependent PTEN expression to regulate malignant progression. Nat. Med..

[293] Catoira M.C., Fusaro L., Di Francesco D., Ramella M., Boccafoschi F. (2019). Overview of natural hydrogels for regenerative medicine applications. J. Mater. Sci. Mater. Med..

[294] Yao Y., Wang P., Li X., Xu Y., Lu G., Jiang Q., Sun Y., Fan Y., Zhang X. (2020). A di-self-crosslinking hyaluronan-based hydrogel combined with type I collagen to construct a biomimetic injectable cartilage-filling scaffold. Acta Biomat..

[295] Zhang Y., Cao Y., Zhao H., Zhang L., Ni T., Liu Y., An Z., Liu M., Pei R. (2020). An injectable BMSC-laden enzyme-catalyzed crosslinking collagen-hyaluronic acid hydrogel for cartilage repair and regeneration. J. Mater. Chem. B.

[296] Chen F., Le P., Fernandes-Cunha G.M., Heilshorn S., Myung D. (2020). Bio-orthogonally crosslinked hyaluronate-collagen hydrogel for suture-free corneal defect repair. Biomaterials.

[297] Lin Y., Chen S., Liu Y., Guo F., Miao O., Huang H. (2023). A composite hydrogel scaffold based on collagen and carboxymethyl chitosan for cartilage regeneration through one-step chemical crosslinking. Int. J. Biol. Macromol..

[298] Prontera C.T., Gallo N., Giannuzzi R., Pugliese M., Primiceri V., Mariano F., Maggiore A., Gigli G., Sannino A., Salvatore L. (2023). Collagen membrane as water-based gel electrolyte for electrochromic devices. Gels.

[299] Shavandi A., Silva T.H., Bekhit A.A., Bekhit A. (2017). Keratin: Dissolution, extraction and biomedical application. Biomater. Sci..

[300] Ye W., Qin M., Qiu R., Li J. (2022). Keratin-based wound dressings: From waste to wealth. Int. J. Biol. Macromol..

[301] Demir G.C., Erdemli E., Keskin D., Tezcaner A. (2022). Xanthan-gelatin and xanthan-gelatin-keratin wound dressings for local delivery of Vitamin C. Int. J. Pharm..

[302] Sadeghi S., Nourmohammadi J., Ghaee A., Soleimani N. (2020). Carboxymethyl cellulose-human hair keratin hydrogel with controlled clindamycin release as antibacterial wound dressing. Int. J. Biol. Macromol..

[303] Leyden J.J., Krochmal L.K., Yaroshinsky A. (2006). Two randomized, double-blind, controlled trials of 2219 subjects to compare the combination clindamycin/tretinoin hydrogel with each agent alone and vehicle for the treatment of acne vulgaris. J. Am. Acad. Dermatol..

[304] Tang A., Li Y., Yao Y., Yang X., Cao Z., Nie H., Yang G. (2021). Injectable keratin hydrogels as hemostatic and wound dressing materials. Biomater. Sci..

[305] Cavallaro G., Caruso M.R., Milioto S., Fakhrullin R., Lazzara G. (2022). Keratin/alginate hybrid hydrogels filled with halloysite clay nanotubes for protective treatment of human hair. Int. J. Biol. Macromol..

[306] Caruso M.R., Cavallaro G., Milioto S., Fakhrullin R., Lazzara G. (2023). Halloysite nanotubes/Keratin composites for wool treatment. Appl. Clay Sci..

[307] Wen Q., Mithieux S.M., Weiss A.S. (2020). Elastin biomaterials in dermal repair. Trends Biotechnol..

[308] Sharma A., Sharma P., Roy S. (2021). Elastin-inspired supramolecular hydrogels: A multifaceted extracellular matrix protein in biomedical engineering. Soft Matter.

[309] Vishwakarma A., Sharpe P., Shi S., Ramalingam M. (2015). An Introduction to Stem Cell Biology and Tissue Engineering in Stem Cell Biology and Tissue Engineering in Dental Sciences.

[310] Mahmood A., Patel D., Hickson B., DesRochers J., Hu X. (2022). Recent progress in biopolymer-based hydrogel materials for biomedical applications. Int. J. Mol. Sci..

[311] Tian D.-M., Wanb H.-H., Chen J.-R., Ye Y.-B., He Y., Liu Y., Tang L.-Y., He Z.-Y., Liu K.-Z., Gao C.-J. (2022). In-situ formed elastin-based hydrogels enhance wound healing via promoting innate immune cells recruitment and angiogenesis. Mater. Today Bio.

[312] Stojic M., Ródenas-Rochina J., López-Donaire M.L., González de Torre I., González Pérez M., Rodríguez-Cabello J.C., Vojtová L., Jorcano J.L., Velasco D. (2021). Elastin-plasma hybrid hydrogels for skin tissue engineering. Polymers.

[313] Raslan A., Saenz del Burgo L., Espona-Noguera A., Ochoa de Retana A.M., Sanjuán M.L., Cañibano-Hernández A., Gálvez-Martín P., Ciriza J., Pedraz J.L. (2020). BSA- and elastin-coated GO, but Not collagen-coated GO, enhance the biological performance of alginate hydrogels. Pharmaceutics.

[314] Griswold E., Cappello J., Ghandehari H. (2022). Silk-elastin like protein-based hydrogels for drug delivery and embolization. Adv. Drug Deliv. Rev..

[315] Radvar E., Azevedo H.S. (2019). Supramolecular peptide/polymer hybrid hydrogels for biomedical applications. Macromol. Biosci..

[316] Erak M., Bellmann-Sickert K., Els-Heindl K., Beck-Sickinger A.G. (2018). Peptide chemistry toolbox—Transforming natural peptides into peptide therapeutics. Bioorg. Med. Chem..

[317] Hollingshead S., Liu J.C. (2020). pH-sensitive mechanical properties of elastin-based hydrogels. Macromol. Biosci..

[318] Nelson D.W., Gilbert R.J. (2021). Extracellular matrix-mimetic hydrogels for treating neural tissue injury: A focus on fibrin, hyaluronic acid, and elastin-like polypeptide hydrogels. Adv. Healthc. Mater..

[319] Chatterjee S., Chi-leung Hui P. (2021). Review of applications and future prospects of stimuli-responsive hydrogel based on thermo-responsive biopolymers in drug delivery systems. Polymers.

[320] Garanger E., Lecommandoux S. (2022). Emerging opportunities in bioconjugates of Elastin-like polypeptides with synthetic or natural polymers. Adv. Drug Deliv. Rev..

[321] Sani E.S., Portillo-Lara R., Spencer A., Yu W., Geilich B.M., Noshadi I., Webster T.J., Annabi N. (2018). Engineering adhesive and antimicrobial hyaluronic acid/elastin like polypeptide hybrid hydrogels for tissue engineering applications. ACS Biomater. Sci. Eng..

[322] Pal P., Nguyen Q.C., Benton A.H., Marquart M.E., Janorkar A.V. (2019). Drug-loaded elastin-like polypeptide–collagen hydrogels with high modulus for bone tissue engineering. Macromol. Biosci..

[323] Luo T., Kiick K.L. (2017). Collagen-like peptide bioconjugates. Bioconjug. Chem..

[324] Ren Y., Zhang H., Qin W., Du B., Liu L., Yang J. (2020). A collagen mimetic peptide-modified hyaluronic acid hydrogel system with enzymatically mediated degradation for mesenchymal stem cell differentiation. Mater. Sci. Eng. C Mater. Biol. Appl..

[325] Chen J., Zou X. (2019). Self-assemble peptide biomaterials and their biomedical applications. Bioact. Mater..

[326] Fan Y., Ren G., Cui Y., Liu H., Li S., Tian Y., Wang G., Peng C., Wang Y., Wu D. (2023). Peptide-based hydrogel for enhanced bone repair. Mater. Des..

[327] Deng A., Yang Y., Du S., Yang X., Pang S., Wang X., Yang S. (2021). Preparation of a recombinant collagen-peptide (RHC)-conjugated chitosan thermosensitive hydrogel for wound healing. Mater. Sci. Eng. C.

[328] López-García G., Dublan-García O., Arizmendi-Cotero D., Gómez Oliván L.M. (2022). Antioxidant and antimicrobial peptides derived from food proteins. Molecules.

[329] Admassu H., Gasmalla M.A.A., Yang R., Zhao W. (2017). Bioactive peptides derived from seaweed protein and their health benefits: Antihypertensive, antioxidant, and antidiabetic properties. J. Food Sci..

[330] Luo Y., Song Y. (2021). Mechanism of antimicrobial peptides: Antimicrobial, anti-inflammatory and antibiofilm activities. Int. J. Mol. Sci..

[331] Mba I.E., Nweze E.I. (2022). Antimicrobial peptides therapy: An emerging alternative for treating drug-resistant bacteria. Yale J. Biol. Med..

[332] Castillo-Juárez I., Blancas-Luciano B.E., García-Contreras R., Fernández-Presas A.M. (2022). Antimicrobial peptides properties beyond growth inhibition and bacterial killing. PeerJ.

[333] Hafeez A.B., Jiang X., Bergen P.J., Zhu Y. (2021). Antimicrobial peptides: An update on classifications and databases. J. Mol. Sci..

[334] Jenssen H., Hamill P., Hancock R.E.W. (2006). Peptide antimicrobial agents. Clin. Microbiol. Rev..

[335] Lee T.H., Hall K.N., Aguilar M.I. (2016). Antimicrobial peptide structure and mechanism of action: A focus on the role of membrane Structure. Curr. Top. Med. Chem..

[336] Kang H.K., Kim C., Seo C.H., Park Y. (2017). The therapeutic applications of antimicrobial peptides (AMPs): A patent review. J. Microbiol..

[337] Zou T.B., He T.P., Li H.B., Tang H.W., Xia E.Q. (2016). The structure-activity relationship of the antioxidant peptides from natural proteins. Molecules.

[338] Wu R.B., Wu C.L., Liu D., Yang X.H., Huang J.F., Zhang J., Liao B., He H.L., Li H. (2015). Overview of antioxidant peptides derived from marine resources: The sources, characteristic, purification, and evaluation methods. Appl. Biochem. Biotechnol..

[339] Nwachukwu I.D., Aluko R.E. (2019). Structural and functional properties of food protein-derived antioxidant peptides. J. Food Biochem..

[340] Sirivibulkovit K., Nouanthavong S., Sameenoi Y. (2018). Paper-based DPPH assay for antioxidant activity analysis. Anal. Sci..

[341] Gulcin I. (2020). Antioxidants and antioxidant methods: An updated overview. Arch. Toxicol..

[342] Forman H.J., Zhang H. (2021). Targeting oxidative stress in disease: Promise and limitations of antioxidant therapy. Nat. Rev. Drug Discov..

[343] Thakkar H., Eerla R., Gangakhedkar S., Shah R.P. (2021). Exploring unexplored biomarkers of oxidative distress and their use. Adv. Redox Res..

[344] Zeng F.S., Yao Y.F., Wang L.F., Li W.J. (2023). Polysaccharides as antioxidants and prooxidants in managing the double-edged sword of reactive oxygen species. Biomed. Pharmacother..

[345] Tang C., Zhou R., Cao K., Liu J., Kan J., Qian C., Jin C. (2023). Current progress in the hypoglycemic mechanisms of natural polysaccharides. Food Funct..

[346] Fernando I.P.S., Jayawardena T.U., Wu J. (2023). Marine proteins and peptides: Production, biological activities, and potential applications. Food Innov. Adv..

[347] Zhu Y., Lao F., Pan X., Wu J. (2022). Food protein-derived antioxidant peptides: Molecular mechanism, stability and bioavailability. Biomolecules.

[348] Sonklin C., Alashi A.M., Laohakunjit N., Aluko R.E. (2021). Functional characterization of mung bean meal protein-derived antioxidant peptides. Molecules.

[349] Xie Z., Wang X., Yu S., He M., Yu S., Xiao H., Song Y. (2021). Antioxidant and functional properties of cowhide collagen peptides. J. Food Sci..

[350] Xia J., Song H., Huang K., Li S., Guan X. (2020). Purification and characterization of antioxidant peptides from enzymatic hydrolysate of mungbean protein. J. Food Sci..

[351] Li X., Guo M., Chi J., Ma J. (2020). Bioactive peptides from walnut residue protein. Molecules.

[352] Wang J., Liu J., John A., Jiang Y., Zhu H., Yang B., Wen L. (2022). Structure identification of walnut peptides and evaluation of cellular antioxidant activity. Food Chem..

[353] Yesiltas B., García-Moreno P.J., Gregersen S., Olsen T.H., Jones N.C., Hoffmann S.V., Marcatili P., Overgaard M.T., Hansen E.B., Jacobsen C. (2022). Antioxidant peptides derived from potato, seaweed, microbial and spinach proteins: Oxidative stability of 5% fish oil-in-water emulsions. Food Chem..

[354] Wang L., Ma M., Yu Z., Du S. (2021). Preparation and identification of antioxidant peptides from cottonseed proteins. Food Chem..

[355] Nirmal N.P., Rajput M.S., Rathod N.B., Mudgil P., Pati S., Bono G., Nalinanon S., Li L., Maqsood S. (2023). Structural characteristic and molecular docking simulation of fish protein-derived peptides: Recent updates on antioxidant, anti-hypertensive and anti-diabetic peptides. Food Chem..

[356] Xiang Z., Xue Q., Gao P., Yu H., Wu M., Zhao Z., Li Y., Wang S., Zhang J., Dai L. (2023). Antioxidant peptides from edible aquatic animals: Preparation method, mechanism of action, and structure-activity relationships. Food Chem..

[357] Shvachkin Y.P., Mishin G.P., Korshunova G.A. (1982). Advances and prospects in the chemistry of nucleoaminoacids and nucleopeptides. Russ. Chem. Rev..

[358] Bandela A.K., Wagner N., Sadihov H., Morales-Reina S., Chotera-Ouda A., Basu K., Cohen-Luria R., de la Escosura A., Ashkenasy G. (2021). Primitive selection of the fittest emerging through functional synergy in nucleopeptide networks. Proc. Natl. Acad. Sci. USA.

[359] Roviello G.N., Oliviero G., Di Napoli A., Borbone N., Piccialli G. (2020). Synthesis, self-assembly-behavior and biomolecular recognition properties of thyminyl dipeptides. Arab. J. Chem..

[360] Li X., Yi K., Shi J., Gao Y., Lin H.-C., Xu B. (2011). Multifunctional, biocompatible supramolecular hydrogelators consist only of nucleobase, amino acid, and glycoside. J. Am. Chem. Soc..

[361] Wang H., Feng Z., Xu B. (2019). Supramolecular assemblies of peptides or nucleopeptides for gene delivery. Theranostics.

[362] Yuan D., Du X., Shi J., Zhou N., Baoum A.A., Xu B. (2014). Synthesis of novel conjugates of a saccharide, amino acids, nucleobase and the evaluation of their cell compatibility. Beilstein J. Org. Chem..

[363] Boback K., Bacchi K., O’Neill S., Brown S., Dorsainvil J., Smith-Carpenter J.E. (2020). Impact of C-terminal chemistry on self-assembled morphology of guanosine containing nucleopeptides. Molecules.

[364] Musumeci D., Roviello V., Roviello G.N. (2018). DNA- and RNA-binding ability of oligoDapT, a nucleobase-decorated peptide, for biomedical applications. Int. J. Nanomed..

[365] Roviello G.N., Musumeci D., Moccia M., Castiglione M., Sapio R., Valente M., Bucci E.M., Perretta G., Pedone C. (2007). dabPna: Design, synthesis, and DNA binding studies. Nucleosides Nucleotides Nucleic Acids.

[366] Roviello G.N., Musumeci D., Pedone C., Bucci E.M. (2010). Synthesis, characterization and hybridization studies of an alternate nucleo-ε/γ-peptide: Complexes formation with natural nucleic acids. Amino Acids.

[367] Musumeci D., Ullah S., Ikram A., Roviello G.N. (2022). Novel insights on nucleopeptide binding: A spectroscopic and in silico investigation on the interaction of a thymine-bearing tetrapeptide with a homoadenine DNA. J. Mol. Liq..

[368] Du X., Zhou J., Li X., Xu B. (2017). Self-assembly of nucleopeptides to interact with DNAs. Interface Focus.

[369] Tomassi S., Ieranò C., Del Bene A., D’Aniello A., Napolitano M., Rea G., Auletta F., Portella L., Capiluongo A., Mazzarella V. (2022). Tailoring the structure of cell penetrating DNA and RNA binding nucleopeptides. Int. J. Mol. Sci..

[370] Tomassi S., Montalban F.F., Russo R., Novellino E., Messere A., Di Maro S. (2019). Investigation of the stereochemical-dependent DNA and RNA binding of arginine-based nucleopeptides. Symmetry.

[371] Noblett A.D., Baek K., Suggs L.J. (2021). Controlling nucleopeptide hydrogel self-assembly and formation for cell-culture scaffold applications. ACS Biomater. Sci. Eng..

[372] Baek K., Noblett A., Ren P., Suggs L.J. (2019). The design and characterization of nucleo-peptides for hydrogel self-assembly. ACS Appl. Bio Mater..

[373] Ghosh S., Ghosh T., Bhowmik S., Patidar M.K., Das A.K. (2023). Nucleopeptide-coupled injectable bioconjugated guanosine-quadruplex hydrogel with inherent antibacterial activity. ACS Appl. Bio Mater..

[374] Wang H., Feng Z., Qin Y., Wang J., Xu B. (2018). Nucleopeptide assemblies selectively sequester ATP in cancer cells to increase the efficacy of doxorubicin. Angew. Chem. Int. Ed..

[375] Scognamiglio P.L., Platella C., Napolitano E., Musumeci D., Roviello G.N. (2021). From prebiotic chemistry to supramolecular biomedical materials: Exploring the properties of self-assembling nucleobase-containing peptides. Molecules.

[376] Giraud T., Hoschtettler P., Pickaert G., Averlant-Petit M.C., Stefan L. (2022). Emerging low-molecular weight nucleopeptide-based hydrogels: State of the art, applications, challenges and perspectives. Nanoscales.

[377] Datta D., Tiwari O., Gupta M.K. (2019). Self-assembly of diphenylalanine–peptide nucleic acid conjugates. ACS Omega.

[378] Murai K., Inagaki K., Hiraoka C., Minoshima S., Kinoshita T., Nagata K., Higuchi M. (2019). Mineralization of magnetic nano-tape in self-organized nanospace composed of nucleopeptides and peptides. Cryst. Eng. Comm..

[379] Immel J.R., Bloom S. (2022). Carba-nucleopeptides (cNPs): A biopharmaceutical modality formed through aqueous Rhodamine B photoredox catalysis. Angew. Chem. Int. Ed..

[380] Ghilan A., Croitoriu A., Chiriac A.P., Nita L.E., Bercea M., Rusu A.G. (2023). Injectable networks based on a hybrid synthetic/natural polymer gel and self-assembling peptides functioning as reinforcing fillers. Polymers.

[381] Rusu A.G., Nita L.E., Simionescu N., Ghilan A., Chiriac A.P., Mititelu-Tartau L. (2023). Enzymatically-crosslinked gelatin hydrogels with nanostructured architecture and self-healing performance for potential use as wound dressings. Polymers.

[382] Constantinou A.P., Georgiou T.K. (2021). Pre-clinical and clinical applications of thermoreversible hydrogels in biomedical engineering: A review. Polym. Int..

[383] Ovrebo O., Perale G., Wojciechowski J.P., Echalier C., Jeffers J.R.T., Stevens M.M., Haugen H.J., Rossi F. (2022). Design and clinical application of injectable hydrogels for musculoskeletal therapy. Bioeng. Transl. Med..

[384] James J.R., Curd J., Ashworth J.C., Abuhantash M., Grundy M., Seedhouse C.H., Arkill K.P., Wright A.J., Merry C.L.R., Thompson A. (2023). Hydrogel-based pre-clinical evaluation of repurposed FDA-approved drugs for AML. Int. J. Mol. Sci..

[385] Karami P., Stampoultzis T., Guo Y., Pioletti D.P. (2023). A guide to preclinical evaluation of hydrogel-based devices for treatment of cartilage lesions. Acta Biomat..

